# A conserved motif in three viral movement proteins from different genera is required for host factor recruitment and cell-to-cell movement

**DOI:** 10.1038/s41598-020-61741-5

**Published:** 2020-03-16

**Authors:** José A. Navarro, Marta Serra-Soriano, Lorena Corachán-Valencia, Vicente Pallás

**Affiliations:** Instituto de Biología Molecular y Celular de Plantas. Consejo Superior de Investigaciones Científicas-Universidad Politécnica de Valencia. Avda. Ingeniero Fausto Elio, 46022 Valencia, Spain

**Keywords:** Virus-host interactions, Biotic

## Abstract

Due to their minimal genomes, plant viruses are forced to hijack specific cellular pathways to ensure host colonization, a condition that most frequently involves physical interaction between viral and host proteins. Among putative viral interactors are the movement proteins, responsible for plasmodesma gating and genome binding during viral transport. Two of them, DGBp1 and DGBp2, are required for alpha-, beta- and gammacarmovirus cell-to-cell movement, but the number of DGBp-host interactors identified at present is limited. By using two different approaches, yeast two-hybrid and bimolecular fluorescence complementation assays, we found three Arabidopsis factors, eIF3g1, RPP3A and WRKY36, interacting with DGBp1s from each genus mentioned above. eIF3g1 and RPP3A are mainly involved in protein translation initiation and elongation phases, respectively, while WRKY36 belongs to WRKY transcription factor family, important regulators of many defence responses. These host proteins are not expected to be associated with viral movement, but knocking out *WRKY36* or silencing either *RPP3A* or *eIF3g1* negatively affected Arabidopsis infection by *Turnip crinkle virus*. A highly conserved FNF motif at DGBp1 C-terminus was required for protein-protein interaction and cell-to-cell movement, suggesting an important biological role.

## Introduction

RNA plant viruses have some of the smallest and compact coding genomes of any organism on Earth. Although this feature allows viruses to tolerate high mutation rates and replicate faster, it also implies that only a few genes fit in their genomes^1,2^. To circumvent their limited coding capacity, plant viruses are forced to take over basic cellular processes, such as protein translation and transport. Plant viruses must also overcome antiviral defence mechanisms, such as post-transcriptional gene silencing, to create a favourable environment that supports their life cycles. How plant viruses can perform all these tasks on their own is still a matter of study. A helping strategy could be provided by the fact that some viral proteins, mainly coat proteins and helper components, are modifiable factors that can recruit multiple pro-viral host proteins enabling them to perform numerous functions in virus life^3,4^. In the same way, they can be targets of antiviral defence factors^5,6^. Therefore, identifying host factors that associate with viral components during infection may provide insights into the virus-host relationships that are essential in regulating the infectious process.

Alpha-, beta- and gammacarmoviruses are among the smallest plant RNA viruses. Their genomes consist of five open reading frames (ORF), most of them overlapping with each other in a unique molecule of single-stranded RNA about 4 kb in length. ORFs included an RNA dependent RNA polymerase, an auxiliary replicase, two small movement proteins (MP), which were initially referred to as the double gene block proteins (DGBp1 and DGBp2), and the coat protein (CP)^7,8^. However, investigation on how these viral factors interact with cellular proteins is scarce and limited to the type member of *Betacarmovirus*, *Turnip crinkle virus* (TCV), especially to its CP. For example, TCV CP mimics the WG/GW motifs found in cellular proteins to physically interact with unloaded Arabidopsis AGO1^9^ or AGO2^10^, suppressing RNA silencing. TCV CP also binds TIP (TCV-interacting protein), a NAC transcription factor, keeping it outside from the nucleus and modifying gene expression to inhibit the basal immune response^11^. In an earlier work by Lin and Heaton^12^, another TCV protein rather than CP was tested looking for plant interactors. Authors performed a yeast two-hybrid (Y2H) library screening using TCV DGBp1 (p8) as bait. However, only an Arabidopsis protein of unknown function initially named as Atp8 was reported to interact with p8.

MPs play a central role in the infection spreading as they bind viral genomes and move them towards plasmodesmata. Once there, MPs facilitate viral invasion of neighbouring cells by contributing to plasmodesma gating^13^. To do this, the DGB-based transport system exploits the co-ordinated action of DGBp1 and DGBp2. Both MPs have been found in members of genera belonging to the family *Tombusviridae*^14,15^. Still, their implication in cell-to-cell movement has only been shown for alpha, beta and gammacarmoviruses, but earlier for betanecroviruses^16–19^.

Structural and molecular studies performed with DGBp1 of *Carnation mottle virus* (CarMV p7) and *Melon necrotic spot virus* (MNSV p7A), the type members of genera *Alpha*- and *Gammacarmovirus*, respectively, showed that their RNA binding capability relied on an α-helical central region^14,20–22^. This property was directly connected to both MNSV and TCV cell-to-cell movement^17,23^. Regarding DGBp1 subcellular localization and dynamics, p7A was localized in cytoplasmic motile granules that associate with actin microfilaments, showed actin-dependent movement and accumulated at the cell periphery^23^. In the case of p7, subcellular fractionation experiments showed that it was mainly associated with the cell wall fraction^24^. Contrary to what could be expected for an MP, ectopically expressed TCV p8 showed a nuclear localization that was required for cell-to-cell movement^25^.

The specific role of DGBp2s in viral transport is still unclear, although some important features have arisen during the last few years. These proteins are characterised by their ability to associate with membranes. CarMV, TCV and MNSV DGBp2s were all targeted and inserted *in vitro* into canine endoplasmic reticulum-derived microsomes^14,26,27^. Besides, MNSV and PFBV DGBp2s were demonstrated to associate with endoplasmic reticulum membranes in plants^18,28^, but only the former was further targeted to plasmodesmata through Golgi in a COPII-dependent pathway^15^.

Since Lin and Heaton work in 2001, it has not been reported any data on host proteins interacting with either DGBp1. In this study, we identified three Arabidopsis factors, eIF3g1, RPP3A and WRKY36, that interacted with MNSV, CarMV and TCV DGBp1 (p7A, p7 and p8, respectively) in two different systems. We also characterised a DGBp1-shared aromatic motif, FNF, at the C-terminal region, as the unique responsible for the interaction. Finally, we showed that depletion of each host factor negatively affects Arabidopsis infection by TCV. Although eIF3g, RPP3A and WRKY36 are not expected to be involved in viral transport, abolishing their interaction to the TCV p8 by FNF mutation to VNV inhibited TCV cell-to-cell movement. Our results expand the varied repertoire of host factors that interact with viral MPs and describe for the first time common candidates for three different virus genera.

## Results

### A highly conserved FNF motif is essential for DGBp1 homo- and heteromerization

**Figure 1:**
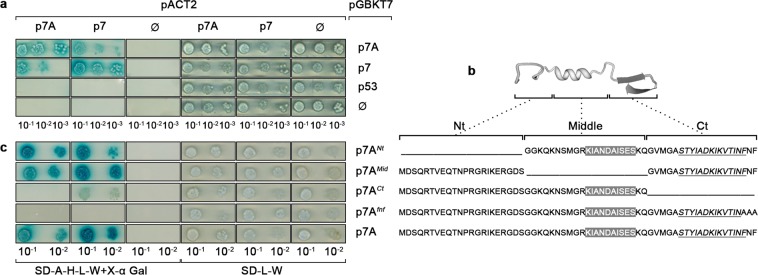
(**a**) Yeast two-hybrid assay showing homo- and hetero-dimerization of p7A and p7. p7A and p7 were fused to either GAL4 Binding Domain or GAL4 Activation Domain in pGBKT7 and pACT2, respectively. As negative controls, pACT2, pGBKT7 and pGB-p53 were used. Different combinations of these vectors were transformed into the yeast strain AH109 as indicated. Yeast diploids were spot-plated in ten-fold serial dilutions to detect the activation of HIS3, ADE2, and MEL1 reporter genes. (Left) SD + DO-A-H-L-W + X-α Gal medium. (Right) SD + DO-L-W medium, serving as a positive control to show equal plating of cells. (**b**) Ribbon diagram showing the p7A-predicted secondary structure and mutants used for mapping the domain involved in self-interaction. The sequences of the predicted α-helix and β-sheet structures are shown in grey boxes and underlined italic font, respectively. (**c**) Y2H for mapping the domain involved in p7A self-interaction. AH109 yeast carrying p7A or each indicated mutant fused to the Gal4 BD in pGBKT7 was transformed with pAC-p7A or pAC-p7. pACT2 was used as control. The assay was performed as indicated before.

In previous work, we showed that MNSV p7A self-interacted both *in vitro* by polyacrylamide gel electrophoresis and *in vivo* by bimolecular fluorescence complementation (BiFC)^23^. Here, we extend the analysis to CarMV DGBp1 (p7). We found that both DGBp1s not only self-interacted but also formed heteromers (Fig. 1a). To define DGBp1 self-interaction domain, we perform a multiple sequence alignment of 27 DGBp1s, which belonged to different genera within the *Tombusviridae* family, looking for conserved elements. As it is shown in Supplementary Fig. S1, DGBp1s displayed low amino acid similarity among them except for a highly conserved C-terminal FNF motif. Besides, the consensus data obtained using seven different prediction methods revealed the presence of two highly conserved elements of secondary structure: an α-helix domain in the middle region, previously related to RNA binding^20,23^, and a potential β-sheet folding at the C-terminal end. Based on these observations and assuming that conserved elements could be functionally or structurally significant, we generated three deletion mutant constructs, pBD-p7A^*Nt*^, pBD-p7A^*Mid*^ and pBD-p7A^*Ct*^ (Fig. 1b). An additional construct in which each residue of the conserved FNF triplet was mutated to alanine, pBD-p7A^*fnf*^, was also obtained. Y2H assays undertaken with pAC-p7A and pAC-p7 in combination with the mutants described above showed that only yeast transformed with constructs pBD-p7A^*Ct*^ or pBD-p7A^*fnf*^ failed to grow (Fig. 1c). These results indicate that the p7A C-terminal region, specially FNF motif, must play an essential role in DGBp1-DGBp1 interaction.

### Identification of common cellular factors interacting with three DGBp1s by Y2H screens

**Figure 2:**
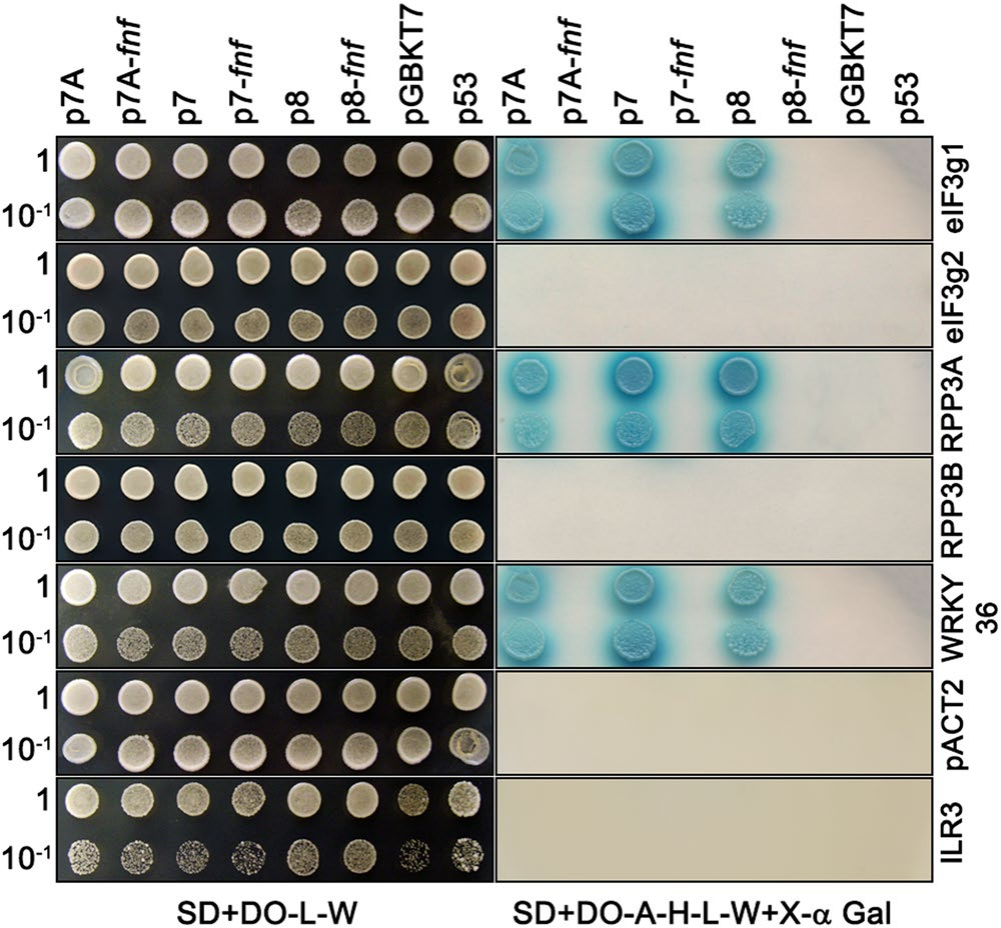
Interaction of p7A, p7 and p8 with eIF3g1, RPP3A and WRKY36 by Y2H. AH109 yeast carrying plasmids expressing p7A, p7, p8 and mutants p7A^*fnf*^, p7^*fnf*^ and p8^*fnf*^ fused to the Gal4-BD was transformed with plasmids expressing WRKY36, eIF3g1, or RPP3A fused to the Gal4-AD. As control, bait strains were transformed with pACT2 or pAD-ILR3 expressing a bLHL transcription factor of *A. thaliana*. A plasmid expressing eIF3g2, or RPP3B fused to the Gal4-AD were also included. Yeast diploids were spot-plated in ten-fold serial dilutions to detect HIS3, ADE2, and MEL1 activation. (Left) SD + DO-L-W medium, serving as a positive control to show equal plating of cells. (Right), SD + DO-A-H-L-W + X-α Gal medium.

To identify new DGBp1-host interacting partners, two independent Y2H screens were conducted with MNSV p7A (*Gammacarmovirus*) and CarMV p7 (*Alphacarmovirus*) as baits and an Arabidopsis cDNA library as prey. We initially identified 18 and 33 potential p7A- and p7-interacting Arabidopsis proteins, respectively, with nine of them being shared (Supplementary Table S1). Since genomic libraries contain random fragments of proteins, a screen will identify not only full-length interactors but also interacting subdomains. It is possible that a domain is capable of interacting with some bait, but not the complete protein when other parts of the protein impede the interaction. Therefore, large-scale library screens probably yield a high number of interactions that do not occur in a physiological context. Considering that most of the sequenced library inserts were partial genes, we recheck all the interactions by Y2H but using full-length ORFs. The complete sequence of each gene showed in Supplementary Table S1 was obtained by BLAST searching at The Arabidopsis Information Resource and cloned in pGBDKT7. We also included TCV p8, as representative of betacarmoviruses (pBD/p8), the *fnf* mutant of each DGBp1 (pBD-p7A^*fnf*^, pBD-p7^*fnf*^ and pBD-p8^*fnf*^) and two non-interactor controls, pGBDKT7 and the human tumour protein p53 (pBD-p53). The transcription factor ILR3 (At5g54680)^29^ fused to the Gal4 activation domain, AD (pAC-ILR3) and pACT2 were used as negative controls to challenge pBD constructs. Seven full-length Arabidopsis proteins reproduced the interaction with at least one of the three DGBp1s on re-testing (Supplementary Table S1 and Fig. 2), but only three interacted with all DGBp1s including p8: the subunit G of the eukaryotic translation initiation factor 3 (eIF3g1, At3g11400), the 60 S acidic ribosomal protein P3 (RPP3A, At4g25890) and the transcription factor WRKY36 (At1g69810). Neither mutant proteins p7A^*fnf*^, p7^*fnf*^ and p8^*fnf*^ nor controls resulted in yeast growth (Fig. 2).

Arabidopsis has two isoforms for eIF3g, eIF3g1 (At3g11400) and eIF3g2 (At5g06000) sharing 65.9% amino acid sequence identity, and two isoforms for RPP3, RPP3A (At4g25890) and RPP3B (At5g57290) sharing 83.3% amino acid sequence identity. To determine whether eIF3g2 and RPP3B could also interact with any of the three DGBp1s, we also included pBD-eIF3g2 or pBD-RPP3B as new baits. None of the DGBp1s interacted with eIF3g2 and RPP3B isoforms, indicating a high specificity in the interaction of the three DGBp1s with both eIF3g1 and RPP3A (Fig. 2).

### Validation of the interactions of p7A, p7 and p8 with eIF3g1, RPP3A and WRKY36 using BiFC assays

**Figure 3:**
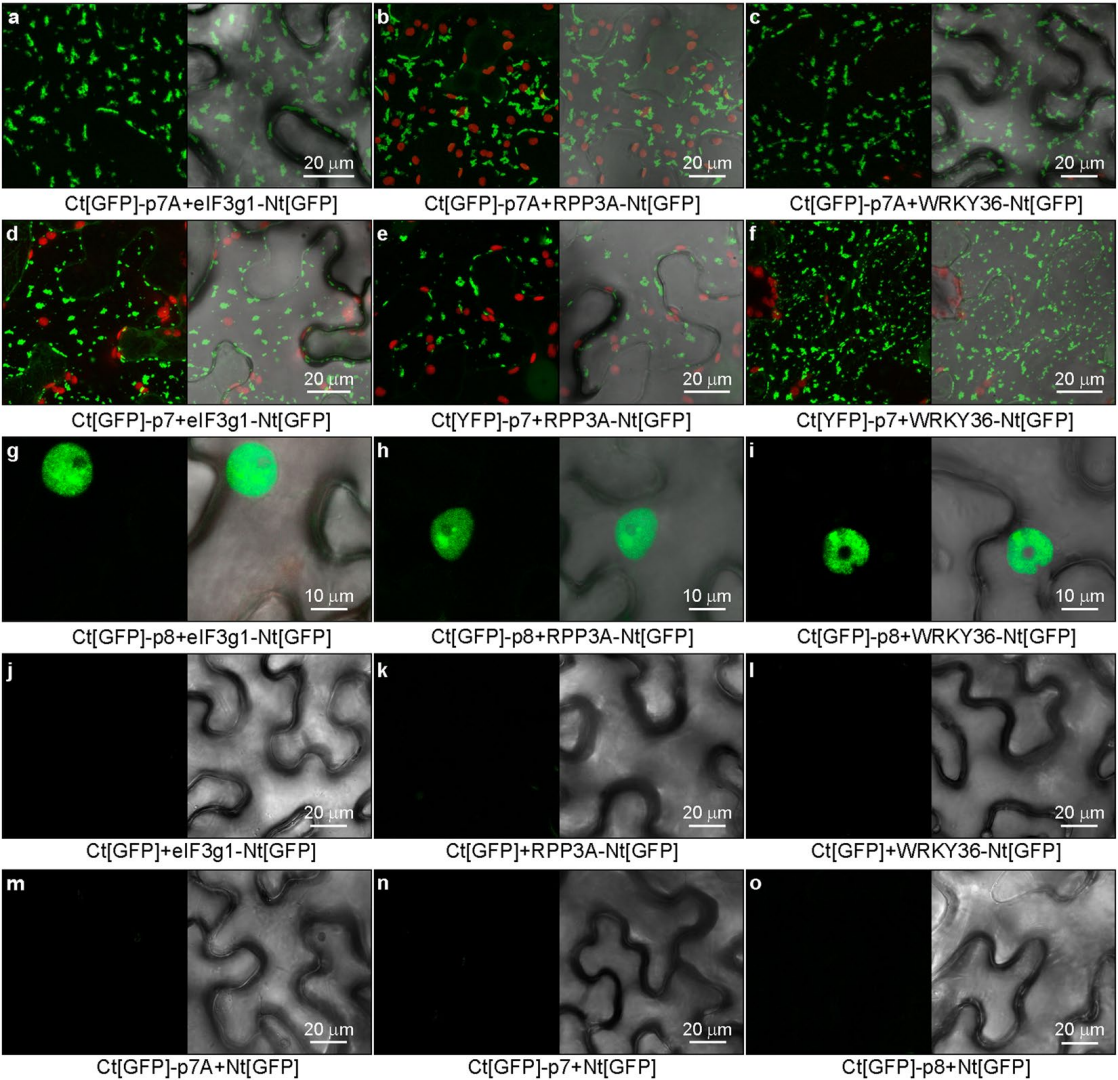
Bimolecular fluorescence complementation assay in epidermal cells of *N. benthamina* using three DGBp1s (p7, p7A and p8) and eIF3g1, RPP3A or WRKY36. Co-expression of eIF3g-N_t_[YFP], RPP3A-Nt[GFP] or WRKY36-Nt[GFP] with Ct[GFP]-p7A (**a**–**c**) or Ct[GFP]-p7 (**d**–**f**) resulted in fluorescent patch-like aggregates while nucleoplasm fluorescence was observed in co-expressions with Ct[GFP]-p8 (**g**–**i**). Co-expression of each construct with its corresponding free Nt[GFP] or Ct[GFP] was used as negative controls (**j**–**o**). Each panel includes a fluorescent Z-series stack projection (left) and its corresponding bright-field overlay image to define cell contour (right) better. The red colour corresponds to chlorophyll fluorescence.

To study whether DGBp1s can also interact with eIF3g1, RPP3A and WRKY36 in plant cells, we also perform BiFC assays. Constructs containing either N-terminal or C-terminal fusions of p7A, p7, p8, eIF3g1, RPP3A or WRKY36 to the N-terminal or C-terminal fragment of GFP (Ct[GFP] or Nt[GFP]) were co-expressed into *Nicotiana benthamiana* leaves in pairwise combinations. To visualize fluorescent emission, epidermal cells were examined under a confocal laser scanning microscope (CLSM) three days after agroinfiltration. Fluorescent signals were seen in combinations including eIF3g1, RPP3A and WRKY36 with Ct[YFP]-DGBp1 or Nt[YFP]-DGBp1 but not in those with DGBp1-Nt[YFP] and DGBp1-Ct[YFP] (Supplementary Table S2 and combinations including Ct[GFP]-DGBp1 are shown in Fig. 3). These findings cannot be attributed to differential expression of the constructs (see Supplementary Fig. S2). Thus, the possibility of steric masking of the FNF motif by Nt[GFP] and Ct[GFP] tags cannot be ruled out. For p7A and p7, all interactions were observed as cytoplasmic patch-like aggregates, most likely compressed in cell periphery by the large central vacuole (Fig. 3a–f and Supplementary Movie S1). In contrast, interactions involving p8 were visualized within the nucleoplasm (Fig. 3g–i). Co-transformation of BiFC constructs of p7A, p7, p8, eIF3g1, RPP3A and WRKY36 with vectors expressing free Ct[GFP] or Nt[GFP] resulted in the absence of fluorescence (Fig. 3j–o and Supplementary Table S2). Therefore, BiFC observations were consistent with Y2H results suggesting the robustness and reliability of the interactions.

### Subcellular localization of the three DGBp1s, eIF3g1, RPP3A and WRKY36

**Figure 4:**
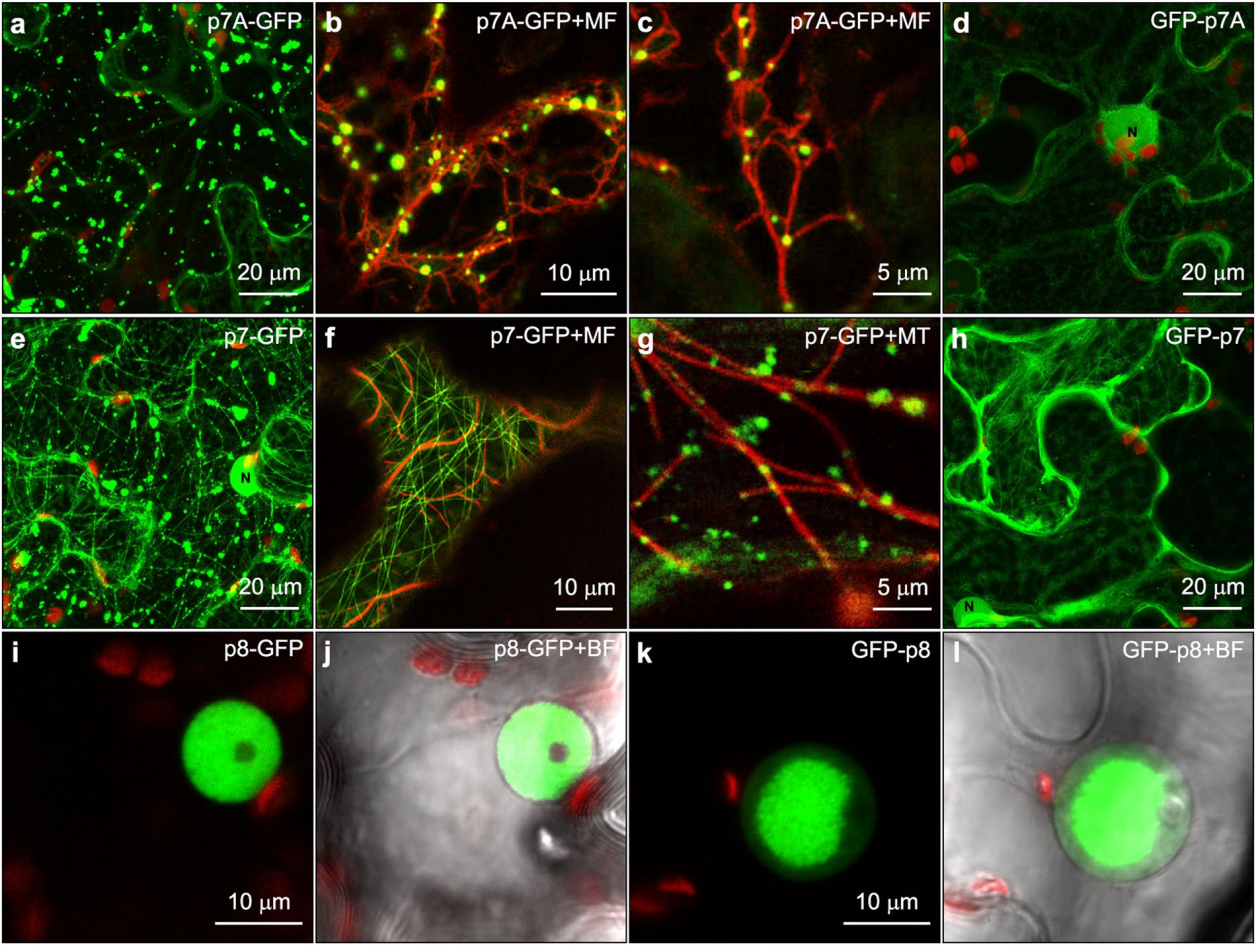
Subcellular localization of DGBp1s ectopically expressed in epidermal cells of *N. benthamiana*. (**a**) Z-stack projection image showing p7A-GFP distribution in cytoplasmic patch-like aggregates. (**b**,**c**) co-expression of p7A-GFP with Talin-dsRed, a red-fluorescence actin microfilament (MF) marker. (**d**) Z-stack projection image showing the nucleocytoplasmic localization of GFP-p7A. (**e**) Z-stack projection image showing p7-GFP distribution in cytoplasmic patch-like aggregates and microtubules. (**f**) Co-expression of p7-GFP with Talin-dsRed in cell region without p7-GFP aggregates to clearly show the difference with microfilaments. (**g**) Co-expression of p7-GFP with αTubulin-ChFP used as red-fluorescence microtubule (MT) marker. (**h**) Z-stack projection image showing the predominant nucleocytoplasmic localization of GFP-p7. (**i**, **k**) Nucleoplasm localization of p8-GFP and GFP-p8, respectively. A large inclusion body is evident in panel k. The corresponding bright-field overlay images (**j**,**l**, respectively) are shown to highlight nucleus contour. Except when indicated images correspond to single scans.

**Figure 5:**
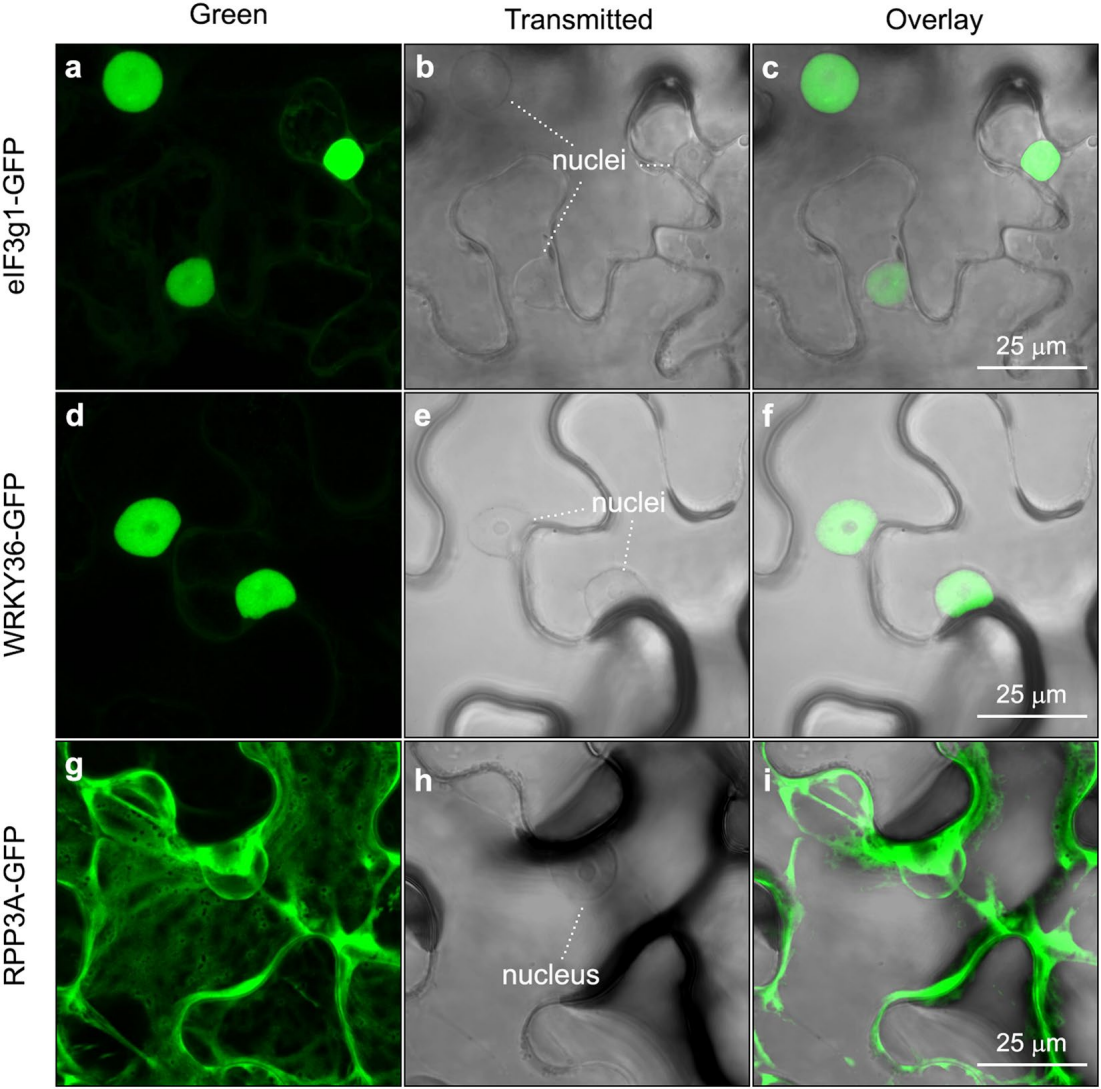
Subcellular localization of eIF3g1-GFP, WRKY36-GFP and RPPA-GFP. Z-stack projection showing the predominant nuclear localization of eIF3g1-GFP and WRKY36-GFP and the nucleus-excluded cytoplasmic distribution of RPP3A-GFP is showed on the left (**a**,**d** and **g**). The transmitted images correspond to single scans where the nuclei were visualised better (**b**,**e** and **h**). The position of the nuclei is indicated. Merged images of green and transmitted channels are shown on the right (**c**,**f** and **i**).

To further assess the consistency of our BiFC findings, we next analysed the subcellular localisation of eIF3g1, RPP3A, WRKY36 and each DGBp1 in plant cells. To do this, the coding sequences of all of them were fused to both the N- and C-terminus of GFP, transiently expressed in leaves of *N. benthamiana* and appropriate expression verified by immunoblotting (Supplementary Fig. S3). As we previously reported, p7A-GFP was distributed in cytoplasmic aggregates, ranging from 1 to 10 µm^2^, that were distributed within the compressed peripheral cytosol (Fig. 4a and Supplementary Movie S2) and associated with microfilaments (highlighted in Fig. 4b,c by dsRFP-Talin, a red-tagged actin-binding protein)^23^. p7-GFP was found in patch-like aggregates, similar in size and location to those observed with p7A-GFP. Besides, p7-GFP was also labelling filamentous structures, which morphologically resembled microtubules, associated with fluorescent aggregates in the majority of cells (Fig. 4e and Supplementary Movie S3). To elucidate the filamentous structure nature, we co-expressed p7-GFP either with dsRFP-Talin or ChFP-α-Tubulin, a red-tagged microtubule component. CLSM imaging revealed that p7-GFP was not associated with microfilaments (Fig. 4f) but with microtubules. In fact, ChFP-α-Tubulin appeared to compete with p7-GFP for microtubule attaching sites, as when coexpressed, p7-GFP was observed in aggregates along ChFP-α-Tubulin-marked microtubules but not labelling them (Fig. 4g). GFP-p7A and GFP-p7 mainly showed a nucleocytoplasmic localization (Fig. 4d–h). In contrast, p8-GFP and GFP-p8 were found in the nucleoplasm as previously reported^25^, but most frequently presenting as a large inclusion body (Fig. 4i–l). In the case of RPP3A, eIF3g1, and WRKY36, their subcellular localization was similar irrespective of the GFP position. eIF3g1 was predominantly targeted to the nucleoplasm with faint staining in the cytosol (eIF3g1-GFP distribution is shown in Fig. 5a–c). As expected for a transcription factor and according to previously published data^30^, WRKY36 was exclusively found in the nucleoplasm (WRKY36-GFP distribution is shown in Fig. 5d–f). In contrast, RPP3A was found in the cytosol but excluded from the nucleus (RPP3A-ChFP distribution is shown in Fig. 5g–i) as previously reported^31^. In fact, a nuclear export signal (ELQRKLVQVSLSAD) was predicted in RPP3A sequence using NetNES^32^ and Wregex^33^ tools. Therefore, the distribution pattern of the reconstituted GFP signal in BiFC assays was quite similar to that of the corresponding DGBp1-GFP.

### Depletion of eIF3g1, RPP3A or WRKY36 negatively affects Arabidopsis Infection by TCV

**Figure 6:**
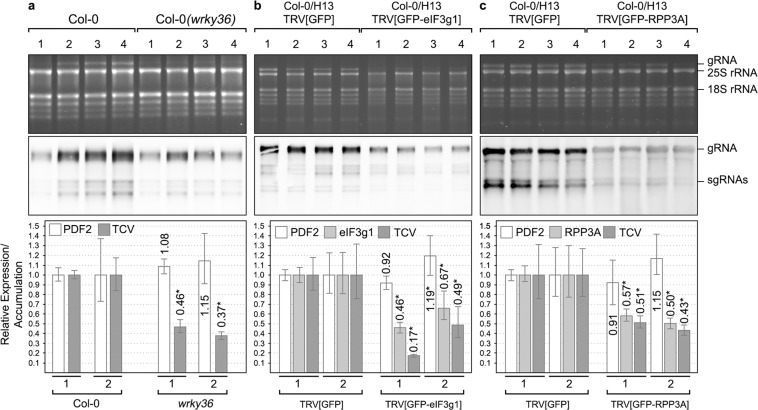
Analysis of turnip crinkle virus accumulation in inoculated leaves of *wrky36* null-mutant (**a**), *eIF3g1*-silenced (**b**) and *RPP3A*-silenced (**c**) Arabidopsis plants. The presence of TCV gRNA and sgRNAs was analysed by Northern blot at 3 dpi (middle panels). The results from a representative assay are shown, but three independent experiments, each one, including ten plants, were performed. Each of the four RNA samples displayed corresponds to a mix of inoculated leaves from 2–3 plants. Ethidium bromide-stained rRNAs serve as loading controls (upper panels). Accumulation of TCV positive-strand gRNA and relative expression of *eIF3g1* and *RPP3A* was analyzed by qRT-PCR in a mix of the four samples by using viral p28 and gene-specific primers, respectively (bottom panels). Relative virus accumulation and gene expression were quantified in two different experiments, as indicated by numbers 1 and 2. The asterisk indicates statistically significant differences (p < 0.05).

**Figure 7:**
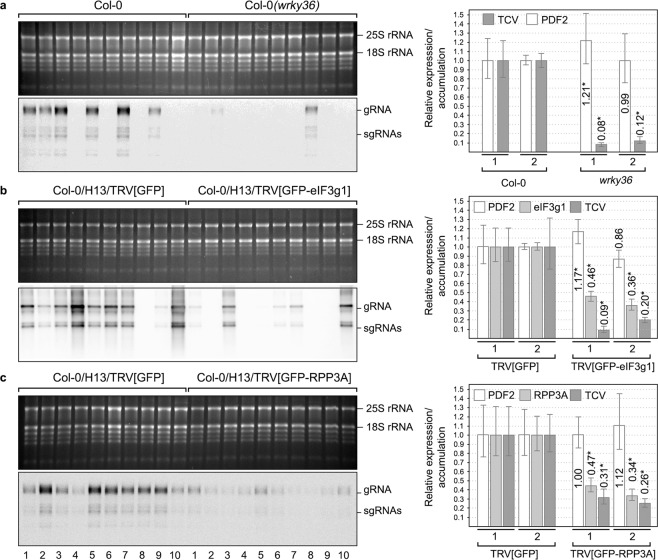
Analysis of turnip crinkle virus accumulation in *wrky36* null-mutant (**a**), *eIF3g1*-silenced (**b**) and *RPP3A*-silenced (**c**) Arabidopsis whole plants without inoculated leaves. The presence of TCV gRNA and sgRNAs was analysed by Northern blot at 3 dpi (middle panels). The results from a representative assay are shown, but three independent experiments, each one including ten plants, were performed. Each of the ten RNA samples displayed corresponds to a unique plant. Ethidium bromide-stained rRNAs serve as loading controls (upper panels). Accumulation of TCV positive-strand gRNA and relative expression of *eIF3g1* and *RPP3A* was analysed by qRT-PCR in a mix of the ten samples by using viral p28 and gene-specific primers, respectively (bottom panels). Relative virus accumulation and gene expression were quantified in two different experiments, as indicated by numbers 1 and 2. The asterisk indicates statistically significant differences (p < 0.05).

TCV is the only one among the three viruses studied here that infects Arabidopsis. Therefore, to investigate to what extent eIF3g1, RPP3A and WRKY36 were involved in virus infection, we used TCV-Arabidopsis system. In the case of WRKY36, we found a T2 mutant with a T-DNA insertion in the exon 3 of *WRKY36* (NASC Code: N108822, SM_3.20017, ecotype Col-0, Supplementary Fig. S4A). The homozygous selection was performed by PCR of genomic DNA following the guidelines from SIGnAL (Salk Institute Genomic Analysis Lab). The absence of full-length mRNA was confirmed by RT-PCR with gene-specific primers (Supplementary Fig. S4). After three-week growth, *wrky36* plants showed no appreciable phenotype (Supplementary Fig. S4). Assays and quantifications were performed as described in Methods. Wild-type Col-0 and *wrky36* plants were inoculated with TCV viral particles, and total RNAs were extracted at 3 days post-inoculation (dpi). TCV RNA accumulation was analysed by Northern blot and qRT-PCR at both local and systemic level. We found a decrease in the hybridisation signal intensities in most of the *wrky36* samples that corresponded to half of the viral accumulation in the inoculated leaves of wild-type plants as quantified by qRT-PCR (p < 0.0001 in assay 1 and p = 0.0014 in assay 2, Fig. 6a). At the systemic level, the vRNA reduction in *wrky36* plants was higher than that observed above most likely due to the added effect of having less vRNA but also fewer infected plants (60% vs 20%) (p < 0.0001 in both assays, Fig. 7a).

We next examined the effect of silencing *eIF3g1* or *RPP3A* on TCV infection, since searching for Arabidopsis knockouts among several T-DNA insertion lines failed. GFP-transgenic Arabidopsis plants (Line H13) were inoculated with TRV1 either plus TRV2[GFP-eIF3g1] (to silence *GFP* and *eIF3g1*), TRV2[GFP-RPP3A] (to silence *GFP* and *RPP3A*) or TRV2[GFP] (to silence *GFP* as control). Approximately ten days later, GFP-fluorescence mostly disappeared in nearly all plants (Supplementary Fig. S5), but only those infected with TRV-[GFP-eIF3g1] showed an apparent phenotype with dwarfing and budding of the youngest leaves (Supplementary Fig S5). Next, we inoculated TCV particles in GFP-silenced leaves and proceeded as above. At the local level, we found a decrease in the TCV hybridisation signal intensities in both eIF3g1 and RPP3A-silenced samples that corresponded to less than a half of the viral accumulation in control samples as quantified by qRT-PCR (p = 0.0028 in eIF3g1 assay 1 and p = 0.0007 in eIF3g1 assay 2, p < 0.0002 in RPP3A assay 1 and p = 0.0008 in RPP3A assay 2, Fig. 6b,c). In general, the more the gene was silenced, the less the virus accumulated (Fig. 6b,c). At the systemic level, a reduction in the number of infected plants was observed when *eIF3g1* (90%) was silenced resulting in a decreasing the total viral RNA quantified (p < 0.0001 in eIF3g1 assay 1 and p = 0.0020 in eIF3g1 assay 2, Fig. 7b). Instead, almost all *RPP3A*-silenced plants were infected but TCV RNA accumulation was considerably reduced compared to control plants (p = 0.0001 in RPP3A assay 1 and p < 0.0001 in RPP3A assay 2, Fig. 7c). As before, gene silencing levels correlated with virus accumulation (Fig. 7b,c).

In contrast to eIF3g1 and RPP3A, that showed a significant reduction of their expression levels in all assays after the infection with the appropriate TRV vector, no significant differences were found in the housekeeping gene PDF2 levels except for local eIF3g1 assay 2, systemic WRKY36 assay 1 and eIF3g1 assay 1. In these latter cases, a slight increase of PDF2 levels was observed (Figs. 6 and 7). No significant differences were also found regarding TCV RNA accumulation (p = 0.75, in assay 1 and p = 0.80, in assay 2) and the number of systemically infected plants (90%) between plants previously infected either with TRV2[GFP] or TRV2[∅] (Supplementary Fig. S6). Overall, these results indicate that eIF3g1, RPP3A and WRKY36 positively modulate Arabidopsis infection by TCV.

### Evaluation of the effect of the FNF to VNV mutation in p8 on TCV infectivity

**Figure 8:**
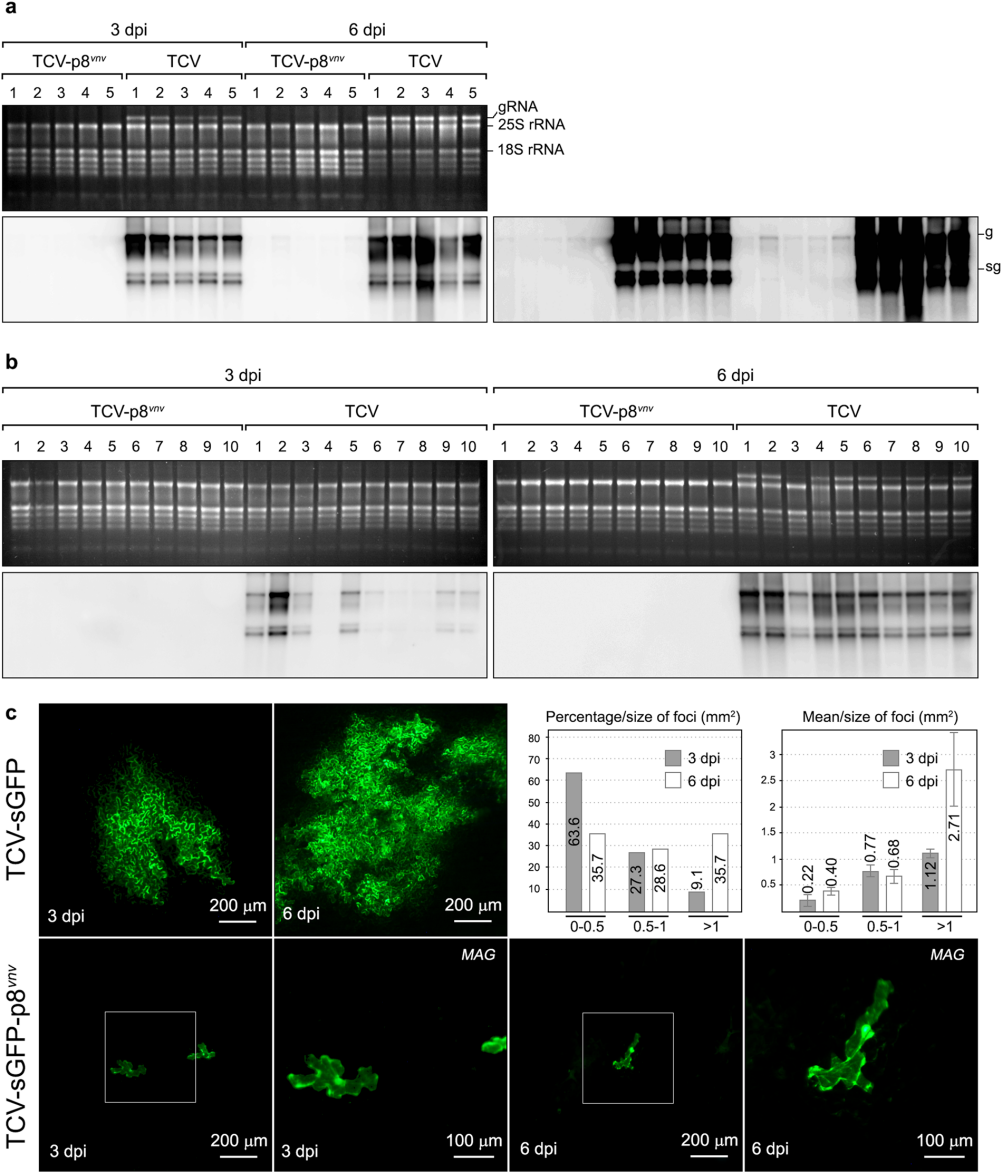
Effect of the FNF to VNV mutation in p8 on TCV infection of Arabidopsis Col-0 plants. (**a**) Northern blot analysis of total RNAs from TCV and TCV-p8^*vnv*^ inoculated leaves at 3 dpi and 6 dpi. The upper panel corresponds to an ethidium bromide-stained gel showing rRNAs as a loading control and TCV gRNA. Bottom panel corresponds to the Northern blot analysis. Overexposure of the same membrane is shown on the right to improve TCV-p8^*vnv*^ RNA detection. Each RNA sample comes from a mix of the inoculated leaves of two plants. The position of TCV gRNA and sgRNAs is indicated. (**b**) Northern blot analysis of total RNAs from TCV and TCV-p8^*vnv*^ upper non-inoculated leaves of the infected plants at 3 and 6 dpi. The upper panels correspond to the ethidium bromide-stained gels showing rRNAs as a loading control and TCV gRNA. Bottom panels correspond to the Northern blot analysis. (**c**) Representative images of TCV-sGFP (upper panels) and TCV-sGFP-p8^*vnv*^ (bottom panels) infection on inoculated leaves were taken by a fluorescence stereomicroscope at 3 and 6 dpi. The bar diagrams on the upper right represent the percentage of TCV-sGFP infection foci grouped by size and the mean size of each group. MAG panels show a magnified image of the region outlined by the square in images on their left.

To further define the role of the FNF motif on TCV infectivity, we analysed the effect of changing the two phenylalanine residues to valine (FNF to VNV) in p8. This mutation was the only one not affecting the overlapping region of the downstream DGBp2 ORF. Moreover, it only required two nucleotide changes that were introduced into both a wild type TCV infectious clone, but also in PZP-TCV-sGFP generating pTCV-p8^*vnv*^ and pTCV-sGFP-p8^*vnv*^, respectively. 20 Arabidopsis Col-0 plants were infected with wild type or mutated RNAs of each TCV variant. As whole plant infection after viral transcript inoculation takes longer than after purified virion inoculation, we analysed both TCV and TCV-p8^*vnv*^ RNA accumulation at 3 and 6 dpi (10 plants per time point). Northern blot revealed that RNAs from TCV-p8^*vnv*^ were barely detected in inoculated leaves at both 3 and 6 dpi even after overexposure of the membranes (Fig. 8a, right panel). In addition, no hybridisation signals were detected from TCV-p8^*vnv*^-infected plants in non-inoculated upper tissue (Fig. 8b), indicating that TCV-p8^*vnv*^ systemic movement was blocked. To better understand what was happening in inoculated leaves, we used TCV-sGFP and TCV-sGFP-p8^*vnv*^ transcripts. Inoculation of these RNAs allows us to monitor the infection by fluorescence appearance since the CP, which is not required for TCV cell-to-cell movement in Arabidopsis^17^, was replaced by GFP. Inoculated leaves from ten plants were analysed at 3 dpi. The majority of infection foci produced by TCV-sGFP (63.6%) showed a mean size smaller than 0.5 mm^2^ (0.22 ± 0.10 mm^2^) and only a 9.1% were bigger than 1 mm^2^ (1.12 ± 0.07 mm^2^) (Fig. 8c). At 6 dpi, TCV-sGFP infection progressed generating infection foci sizes over 0.5 mm^2^ (64.28% of cases), but most of them were bigger than 1 mm^2^ (35.7% of cases, 2.71 ± 1.09 mm^2^) (Fig. 8c). In contrast, FNF mutation in TCV-sGFP-p8^*vnv*^ inhibited virus cell-to-cell movement, since all the infection foci observed were unicellular at both 3 and 6 dpi (Fig. 8c). Together, all findings showed here confer a great functionality to the FNF motif in viral infection.

## Discussion

Molecular basis of plant virus-host relationship has been lately driven by the identification of an increasing number of host factors interacting with viral proteins which can ultimately facilitate or prevent viral infection^4–6^. Here, we report the interaction between three related DGBp1s and three Arabidopsis factors: eIF3g1, RPP3A, and WRKY36. DGBp1s included CarMV p7, TCV p8 and MNSV p7A, each one belonging to the type member of genera *Alpha*-, *Beta*- and *Gammacarmovirus*, respectively. First, we used p7A and p7 to screen an Arabidopsis cDNA library by Y2H looking for common interactors and, in an additional re-evaluation, we also included p8. In all DGBp1s, an FNF conserved motif at their Ct ends was required for all the interactions. We further confirmed these findings by BiFC, but only when the DGBp1 fusions were made downstream of the GFP fragments indicating the involvement of the Ct end in the interaction again. Fluorescence reconstitution, in the case of p7A and p7, resulted in cytoplasmic patch-like aggregates patterns resembling those generated by DGBp1-GFP fusions, that were associated with the cytoskeleton. MPs are involved in viral transport from replication sites to plasmodesmata and from there to adjacent cells. Accordingly, they have been found associated with the cytoskeleton, which they use as the driven force, cellular periphery or plasmodesmata^13^. In previous work, we showed that p7A-GFP failed to form aggregates after Latrunculin B treatment, a compound that inhibits actin polymerization and disrupts actin rearrangement, showing then a nucleocytoplasmic localization^23^. Therefore, the association of p7A and most likely p7 to cytoskeleton favours their aggregation. This association could be hindered by the presence of N-terminal tags as large as GFP (27 kDa) in GFP-p7A/p7 fusions but not by smaller ones such as Nt[GFP] (17.5 kDa) and Ct[GFP] (9.5 kDa) in BiFC constructs.

In the case of p8, the fluorescence reconstitution was observed in the nucleus similarly to its two GFP fusions. However, it is known that TCV cell-to-cell movement needs the binding of p8 to the viral RNA genome^17,34,35^, and this can only happen if p8 is also located in the cytoplasm at least during infection. In fact, an MP is often involved in extensive interactions not only with host proteins but also with the rest of the viral factors, especially in viral genera with several MPs^13^. Additional research is needed to confidently show where DGBp1 interactions take place during viral infection. Nevertheless, considering BiFC just as a Y2H alternative approach to show protein-protein interaction, the consistency among the results obtained with the two different techniques used in this work supports our findings.

As already mentioned above, we also determined the relevance of the FNF motif in self-interaction, interaction with other DGBp1s and with host factors but also in TCV infectivity. The involvement of the C-terminal domain in host factor interaction matches with previous results obtained by Lin and Heaton^12^. They showed that the C-terminal portion of p8 was required for interaction with Atp8. This protein was later identified as RCD1 (RADICAL-INDUCED CELL DEATH1), a nuclear protein that in stress conditions is also localized in the cytoplasm and modulates abscisic acid (ABA), ethylene, and methyl jasmonate responses^36^. Interestingly, RCD1 belongs to the WWE protein-protein interaction domain protein family having a globular domain with three highly conserved residues, two aromatic tryptophan and one glutamic acid residues^37^. Aromatic residues such as phenylalanine, tryptophan, tyrosine and histidine are usually involved in noncovalent interactions between planar aromatic rings, referred to as π-π interactions or π-stacking, that play an essential role in global conformational stability, molecular recognition and self-assembly^38,39^. Although none of the three Arabidopsis factors was expected to be involved a priori in the viral movement, the results show that to viral movement, TCV cell-to-cell and, consequently, systemic movement were blocked when the aromatic residues in the FNF motif were changed to valines. We cannot rule out the possibility that DGBp1s might play relevant roles in different viral cycle steps rather than movement as it has been reported for other MPs^40–42^. In any case, the results reported here confer to the FNF motif a relevant biological functionality.

Our findings also suggest that eIF3g1, RPP3A and WRKY36 could positively regulate viral infection since their depletion reduced viral accumulation in Arabidopsis. As obligate parasites, plant viruses are forced to subvert host translation machinery to translate their messengers. Therefore, it is not surprising that two of the three DGBp1-interactors identified here were ribosome-associated proteins. The large subunit of ribosomes has a lateral protrusion or ribosomal stalk composed of acidic proteins P0, P1, and P2 and the plant-specific P3 (RPP3). P-protein complex is thought to assist in the elongation phase of protein translation and, in maize seedling, alterations of the ribosomal P-protein complex may be involved in the selective translation of mRNA in flooded roots^43,44^. P0 was also reported to act as an extra-ribosomal protein that regulates viral RNA functions in potato virus A infection by promoting viral translation together with eIF(iso)4E and the viral protein VPg^45^. Since DGBp1s are also viral RNA interacting proteins, we can hypothesize that its association with RPP3 could favour specific translation of viral RNAs.

On the other hand, eIF3g is a component of the eukaryotic translation initiation factor 3 (eIF-3) complex that is also required for scanning resumption of post-termination ribosomes, and together with eIF3i stimulates canonical linear scanning. eIF3g can be regarded as an effective target to control host protein translation. DGBp1 interaction with eIF3g could potentially sequester eIF3g, preventing the translation of specific messengers. In this sense, the nucleocapsid of *Measles virus* shuts off host protein translation through eIF3g interaction in mammalian cells, while the selective translation of viral mRNA still occurs^46^. Alternatively, eIF3g could mediate 3′–5′ UTR interactions with the 40 S subunit to facilitate cap-independent translation as recently observed for barley yellow dwarf viral mRNA^47^. Besides, the *Cauliflower mosaic virus* (CaMV) transactivator (TAV) favours the recruitment of the 60 S ribosomal subunit interacting with both eIF3g and 60 S ribosomal protein L24 to promote translation re-initiation of CaMV polycistronic mRNAs^48,49^. Besides, CaMV P6 interacts with plant L18 and L24 ribosomal proteins and initiation factor eIF3 to promote the translation reinitiation of polycistronic viral RNAs^50^. Although genomic expression of alpha-, beta- and gammacarmovirus largely differs from that CaMV since they only produce a bicistronic sgRNA1 for expressing both MPs^51^, the interaction of DGBp1 with eIF3g and RPP3·could also represent a feedback mechanism that modulates MP expression.

Most WRKY transcription factors studied in plant-virus interactions have a significant role in defence responses. Thus, WRKY1 in tobacco and CaWRKYa/d in chilli pepper were involved in the induction of cell death, while WRKY8 in Arabidopsis mediated both abscisic acid (ABA) and ethylene signalling^52–54^. Silencing of WRKY1 in *N. benthamiana* caused lethal apical necrosis and allowed an increase in PVX RNA accumulation^55^, the upregulation of WRKY61 ameliorated viral symptoms after TCV infection^56^, the WRKY30 works as a positive regulator in plant CMV resistance process^57^ and WRKY42 and WRKY80 served as positive regulators in the defence from tomato yellow leaf curl virus infection process in tomato^58^. In this last pathosystem, it was also observed a pro-viral effect of WRKY41 and WRKY54, indicating that WRKY factors can have positive and negative expression patterns in plant-virus interactions. Experimental evidence for WRKY36 function in defence is limited to studies of gene expression profiling. In this sense, WRKY42, WRKY36, and several WRKY group IIIb genes are rapidly induced, with maximal expression 2 h after salicylic acid (SA) application^59^. Moreover, Y2H assays revealed that AtWRKY36 interacted with AtWRKY60 and AtWRKY40, two negative regulators of ABA signalling that, coordinately with WRKY18, modulate plant defence against bacteria and fungi^60^. WRKY36 is also upregulated in response to reactive oxygen and nitrogen species, such as ozone^61^, hydrogen peroxide^62^ and nitric oxide^63^. The two formers accumulate in defence responses against plant-virus^64^, while the last is an important signalling molecule needed for a proper ozone response^65^. Overall, these findings suggest a putative role for WRKY36 in response to plant virus infection.

In summary, the results presented here demonstrate that DGBp1s of alpha- beta- and gammacarmovirus interact with eIF3g1, RPP3A and WRKY36, and that interaction could have a positive effect at least on TCV viral infection. Unravelling the precise role of these host factors could provide not only a deeper understanding of the viral life cycle and valuable new targets to generate viral broad-spectrum resistance, but also useful insights into the functioning of cellular processes, such as molecular trafficking, defence response and plant development.

## Methods

### Molecular cloning for Y2H assays

p7A, p7 and p8 genes were PCR amplified with specific oligonucleotides (Supplementary Table S3) and plasmids pMNSV-Al^16^, pCarMV(Dixie)^20^ and pTCV-M^66^, respectively. Next, cDNAs were cloned into pGBKT7 and pACT2 using the indicated restriction enzymes (Supplementary Table S3) to generate fusions to the Ct end of both the GAL4 binding domain (BD) and GAL4 activation domain (AD), respectively. pBD and pAC are used to denote constructs made with pGBKT7 and pACT2, respectively. pBD-p7A^*Nt*^, pBD-p7A^*Mid*^ and pBD-p7A^*Ct*^ deletion mutants lacking either amino acid residues 1 to 22, 23 to 44, or 45 to 65, respectively, were obtained by inverse PCR using pBD-p7A. pBD-p7A^*fnf*^, pBD-p7^*fnf*^ and pBD-p8^*fnf*^, having the terminal FNF triplet mutated to alanine, were obtained by introducing the mutations into reverse oligonucleotides (Supplementary Table S3). p7A and p7 Arabidopsis-interacting proteins obtained in Y2H screening were identified by BLAST searching at The Arabidopsis Information Resource. Full-length ORFs were obtained using Arabidopsis poly(A) messenger RNAs and SuperScript III One-Step RT-PCR System with Platinum Taq High Fidelity. cDNAs were cloned into pGADT7 using appropriate restriction enzymes and oligonucleotides (Supplementary Table S3).

### Y2H screening

Y2H screening was performed using the GAL4-based MATCHMAKER Two-Hybrid System. For library screening, recombinant bait vectors, pBD-p7A and pBD-p7 were used to transform yeast strain AH109 by the TRAFO protocol^67^. Transformant cells were selected by culturing on SD + DO-W. As prey, we used an Arabidopsis cDNA library cloned into pACT2^68^. Library plasmids were amplified in *E. coli* and purified. Yeast cells harbouring recombinant bait plasmids were transformed with the Arabidopsis library by large-scale transformation protocol. Positive interactions were selected by culturing yeast on SD + DO-A-H-L-W. The α-galactosidase activity (*MEL1*) was analysed by transferring growing colonies to SD + DO-A-H-L-W + X-α Gal plates. Plasmids were isolated following a phenol-chloroform protocol, and redundant pAC/library inserts were identified by restriction fragment length polymorphism after PCR and BsuRI restriction.

Arabidopsis full-length ORFs were cloned in pACT2 and transformed into AH109 yeast cells containing pBD-p7A, pBD-p7A^*fnf*^, pBD-p7, pBD-p7^*fnf*^, pBD-p8 or pBD-p8^*fnf*^. The interactions were re-analysed by culturing co-transformants on SD + DO-A-H-L-W + X-α Gal. To confirm interaction specificity, pACT2 vectors containing the Arabidopsis genes were also transformed in yeast cells harbouring pGBKT7 or pBD-p53. Besides, pGBKT7 vectors containing DGBp1 genes were also transformed in yeast cells harbouring pACT2 or pAC-IL3.

For p7A interaction domain identification, yeast cells were transformed with pBD-p7A, pBD-p7A^*fnf*^, pBD-p7A^*Nt*^, pBD-p7A^*Mid*^, pBD-p7A^*Ct*^ or pBD-p7, and spread on SD + DO-W plates. Next, colonies were grown in SD + DO-W liquid media, pooled and transformed either with pAC-7A or pAC-p7. Co-transformant cells were selected in SD + DO-L-W plates. Positive interactions were analysed as before in SD + DO-A-H-L-W + X-α Gal.

### Molecular cloning for subcellular localization studies and BiFC assays

For subcellular localization studies, p7A, p7, p8, RPP3A, eIF3g1, and WRKY36 cDNAs were fused *in-frame* to the GFP 5′ or 3′ ends. For BiFC assay, both a GFP amino-terminal fragment (1–155, Nt-[GFP]) and a carboxyl-terminal fragment (156–238, Ct-[GFP]) were either fused to the amino or carboxyl terminus of p7A, p7, p8, RPP3A, eIF3g1, and WRKY36 cDNAs. Next, recombinant cDNAs were inserted between CaMV 35 S promoter and PoPit terminator into pMOG800^69^ by using the oligonucleotides and restriction enzymes listed in Supplementary Table S3.

### Agrobacterium tumefaciens-mediated transient expression and bimolecular fluorescence complementation (BiFC) assays

Transient expression assays in *N. benthamiana* were performed as previously described^16^. Briefly, binary constructs were introduced into *Agrobacterium tumefaciens* strain C58C1 by electroporation. Transformed bacteria were grown overnight in Luria-Bertani medium with appropriate antibiotics. Cultures were collected by low-speed centrifugation and adjusted to an OD600 of 0.2 with 10 mM MgCl_2_, 10 mM MES pH 5.6 and 150 µM acetosyringone. These suspensions were introduced in two-week-old leaves of *N. benthamiana* by gentle pressure infiltration into the abaxial side. For experiments requiring the simultaneous expression of two different proteins, bacterial cultures containing the corresponding binary vectors were mixed before infiltration. Plants were kept in growth chambers under long-day photoperiods (16 h light at 25 °C and 8 h dark at 22 °C).

### Confocal laser scanning microscopy and fluorescent markers

Subcellular localization studies and BiFC imaging was conducted with an inverted Zeiss LSM 780 confocal microscope. The fluorescence of eGFP and ChFP/dsRFP was visualized by 488 and 561 nm laser excitation, respectively. The corresponding emission detection windows were 492–532 and 590–630, respectively. The chlorophyll excitation wavelength was 488 nm and fluorescence was detected above 700 nm. The fluorescent organelle markers used were: talin protein fused to dsRFP (dsRFP-Talin) as actin microfilament marker and α-tubulin fused to ChFP (αTubulin-ChFP) as microtubule marker.

### Image analysis

Image analysis was performed using FIJI software. Hybridisation intensity signal was measured on files from Fujifilm LAS-3000 Imager using Fujifilm Image Gauge V4.0.

### Viral induced gene silencing in *A. thaliana*

For VIGS, pTRV1 and pTRV2 Gateway vectors^70^ were used. Fragments of about 300 and 200 bp for eIF3g1 and RPP3A transcripts, respectively, were PCR amplified with gene-specific oligonucleotides (Supplementary Table S3) from Arabidopsis RNAs. Besides, a GFP fragment of 300 bp was amplified from Arabidopsis Line H13 RNAs (NASC Code: N799368) that constitutively express an endoplasmic reticulum resident mGFP5-HDEL. The assembling of GFP-eIF3g1 and GFP-RPP3A fragments was performed by overlap extension PCR and then recombined with pDONR207. The resultant pENTRY vectors were recombined with pTRV2 (pTRV2[GFP-eIF3g1] and pTRV2[GFP-RPP3A]). The target region of each gene was selected using the SGN VIGS Tool^71^. A pTRV2 carrying the complete mGFP5 gene, pTRV2[GFP], was obtained to be used as control. Oligonucleotides used are listed in Supplementary Table S3. Before infiltration, bacteria carrying pTRV1 and pTRV2 (or pTRV2 derivates) were mixed in 1:1 volume ratio (OD600 = 1). 4–5 days after infiltration, a crude extract was made by grinding the infiltrated leaves in inoculation buffer (30 mM sodium phosphate buffer pH 8.0, 20 mM β-Mercaptoethanol) and used to infect two-week-old Line H13 seedlings. GFP silencing was monitored by means of a fluorescence stereomicroscope as from ten days after TRV inoculation. Plants were grown under long-day photoperiods (16 h light at 25 °C and 8 h dark at 22 °C)

### Turnip crinkle virus infection assays

To obtain a virion stock, turnip crinkle virus strain M, transcripts, were synthesized *in vitro* using a cDNA clone provided by Dr Anne Simon. Transcripts were quantified and used to infect two-week-old Arabidopsis (Col-0). Two weeks later, symptomatic leaves were harvested and virions purified by polyethyleneglycol (MW 20000) precipitation as previously described^72^. Site-directed mutagenesis using fully overlapping oligonucleotides (Supplementary Table 3) was used to change the p8 FNF motif to VNV in the above TCV construct but also in PZP-TCV-sGFP plasmid^73^ provided by Dr Steven A. Lommel. The resulting plasmids were named pTCV-p8^*vnv*^ and pTCV-sGFP-p8^*vnv*^, respectively. The virion stock (1 mg/ml) was used for all TCV infection assays, except for those including TCV-p8^*vnv*^ and TCV-sGFP-p8^*vnv*^. In these cases, wild type and mutated transcripts were obtained and directly rubbed on leaves (0.5 µg/leaf). Arabidopsis plants were grown under long-day photoperiods as described above. Approximately three weeks after germination, *wrky36* knock-out (NASC Code: N108822, SM_3.20017) and Arabidopsis TRV-infected plants were mechanically inoculated with TCV virions or transcripts. After three days, total RNA from virion-inoculated leaves and whole plants (without inoculated leaves) was isolated with RiboZol RNA Extraction Reagent. For systemic infection analysis, ten whole plants from three independent assays were analysed (30 samples). For local infection analysis, virion-inoculated leaves from 2–3 different plants were pooled with each assay generating 4 biological replicates (12 samples). When TCV and TCV-p8^*vnv*^ RNAs were inoculated, the samples were taken at 3 and 6 days after inoculation. Isolated RNA (0.4 µg/sample) was analysed by Northern blot hybridisation using a digoxigenin-labelled riboprobe against the TCV CP gene as previously described^74^. In the case of TCV-sGFP and TCV-sGFP-p8^vnv^ RNAs, GFP fluorescence was visualized at 3 and 6 days after inoculation using a Leica MZ16 fluorescence stereomicroscope. Size of infection foci was measured as previously reported^75^.

### Real-time quantitative reverse transcription

DNase I treatment was performed to remove genomic DNA from RNA samples. First-strand cDNA was synthesized from 0.5 µg of total RNA using RevertAid H Minus Reverse Transcriptase and specific oligonucleotides. qRT-PCR was carried out with the ABI 7500 Fast Real-Time PCR detection system using PyroTaq EvaGreen qPCR Supermix, specific oligonucleotides and recommended qPCR cycles. Specific oligonucleotides were designed using Primer3Web 4.1.0^76^. Oligonucleotide efficiencies were verified by qRT-PCR using tenfold serial dilutions of the corresponding cDNA. Each biological replicate consisting of a pool of several independent samples (four samples for local infection and ten samples for systemic infection) was run in triplicate and the entire experiment repeated twice. Two reference genes encoding the elongation factor 1-α (*EF1*, At5g60390) and F-BOX family protein (*F-BOX*, At5g15710) were used to normalize the expression levels^77^. A third reference gene, the protodermal factor 2 (*PDF2*, At1g13320) was used to verify that the VIGS target genes were specifically knocked down, without affecting the expression of other genes^77^. The samples from TRV[GFP] infected plants were used as control. An unpaired t-test was applied to ΔCt values using GraphPad Prism 6 (p < 0.05).

## Supplementary information

Supplementary information.

Supplementary information2.

Supplementary information3.

Supplementary information4.
