# Supplementary material for: A conserved motif in three viral movement proteins from different genera is required for host factor recruitment and cell-to-cell movement
Source: Sci Rep. 2020 Mar 16;10:4758. doi: 10.1038/s41598-020-61741-5 (PMC7075923; doi:10.1038/s41598-020-61741-5)
Supplement: Supplementary file 1 — Supplementary information. [file 41598_2020_61741_MOESM1_ESM.docx]

**SUPPLEMENTARY INFORMATION**

**A conserved motif in three viral movement proteins from different genera is required for host factor recruitment and cell-to-cell movement**

José A. Navarro, Marta Serra-Soriano, Lorena Corachán-Valencia and Vicente Pallás

Instituto de Biología Molecular y Celular de Plantas. Consejo Superior de Investigaciones Científicas-Universidad Politécnica de Valencia. Avda. Ingeniero Fausto Elio, 46022 Valencia, Spain.

**
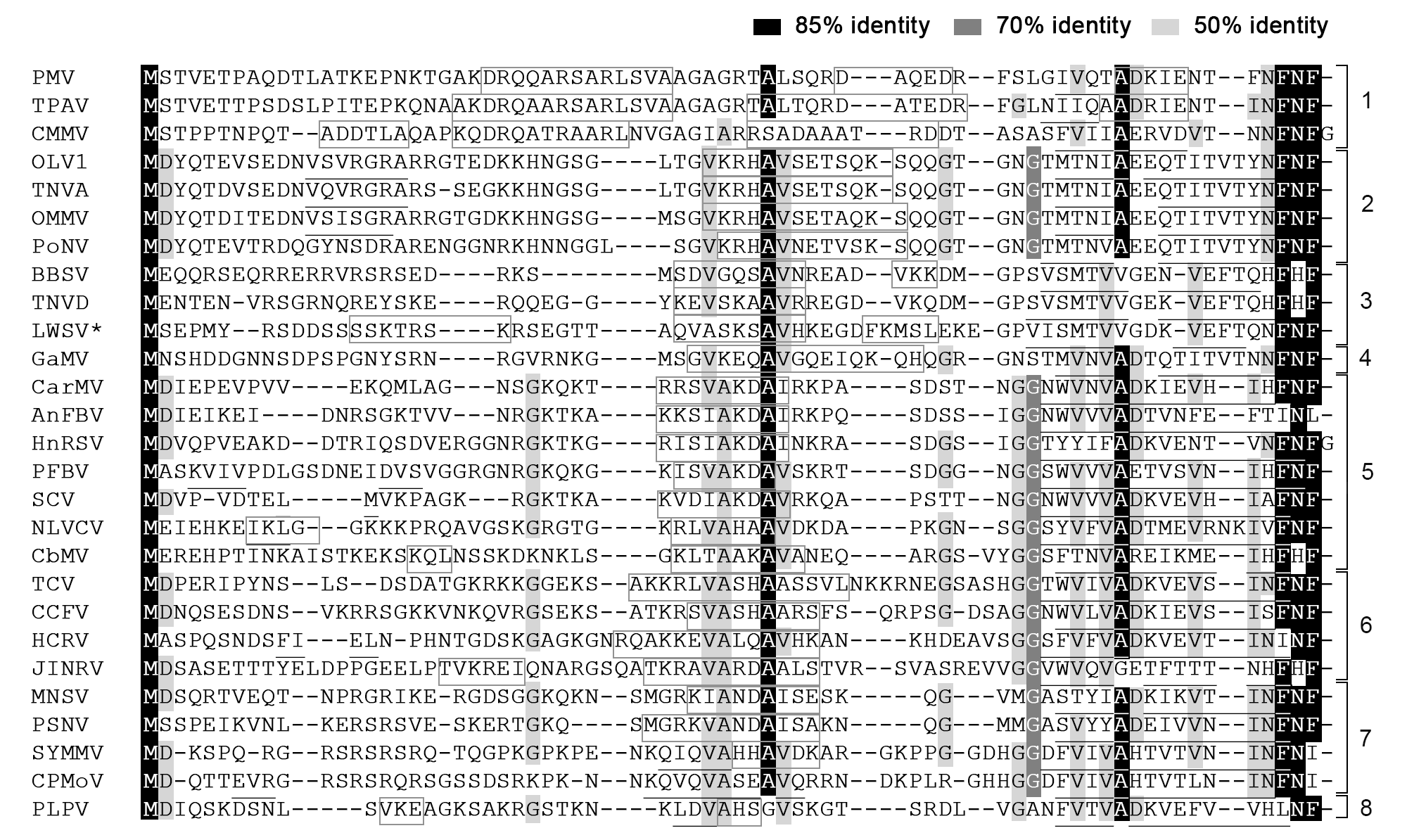
SUPPLEMENTARY FIGURES**

**Supplementary Figure S1.** DGBp1 amino acid sequence alignment of several genus members in family *Tombusviride*. Identity scores are as indicated by colour code. Gaps are represented as contiguous dashes. Amino acid residues predicted to be involved in α-helix are boxed whereas β-sheet structured domains are underlined. 1. Genus *Panicovirus*: PMV, *Panicum mosaic virus*; TPAV, *Thin paspalum asymptomatic virus*; CMMV, *Cocksfoot mild mosaic virus*. 2. Genus *Alphanecrovirus*: OLV1, *Olive latent virus 1*; TNVA, *Tobacco necrosis virus A*; OMMV, *Olive mild mosaic virus*; PoNV, *Potato necrosis virus*. 3. Genus *Betanecrovirus*: BBSV, *Beet black scorch virus*; TNVD, *Tobacco necrosis virus D*; LWSV, *Leek white stripe virus*. 4. Genus *Gallantivirus*: GaMV, *Galinsoga mosaic virus*. 5. Genus *Alphacarmovirus*: CarMV, *Carnation mottle virus*; AnFBV, *Angelonia flower break virus*; HnRSV, *Honeysuckle ringspot virus*; PFBV, *Pelargonium flower break virus*; SCV, *Saguaro cactus virus*; NLVCV, *Nootka lupine vein clearing virus*; CbMV, *Calibrachoa mottle virus*. 6. Genus *Betacarmovirus*: TCV, *Turnip crinkle virus*; CCFV, *Cardamine chlorotic fleck virus*; HCRV, *Hibiscus chlorotic ringspot virus*; JINRV, *Japanese iris necrotic ring virus*. 7. Genus *Gammacarmovirus*: MNSV, *Melon necrotic spot virus*; PSNV, *Pea stem necrosis virus*; SYMMV, *Soybean yellow mottle mosaic virus*; CPMoV, *Cowpea mottle virus*. 8. Genus *Pelarspovirus*: PLPV, *Pelargonium line pattern virus*. Sequence alignment and secondary structure prediction. Amino acid sequences were aligned by using MEGA 7.0.21. The consensus secondary structure was obtained using seven different computational prediction methods (PHDpsi, PROFsec, SSPro 2.01, Predator, YASPIN, JNet and PSIPred) available on the SYMPRED prediction server (http://ibivu.cs.vu.nl/programs/sympredwww/).

**
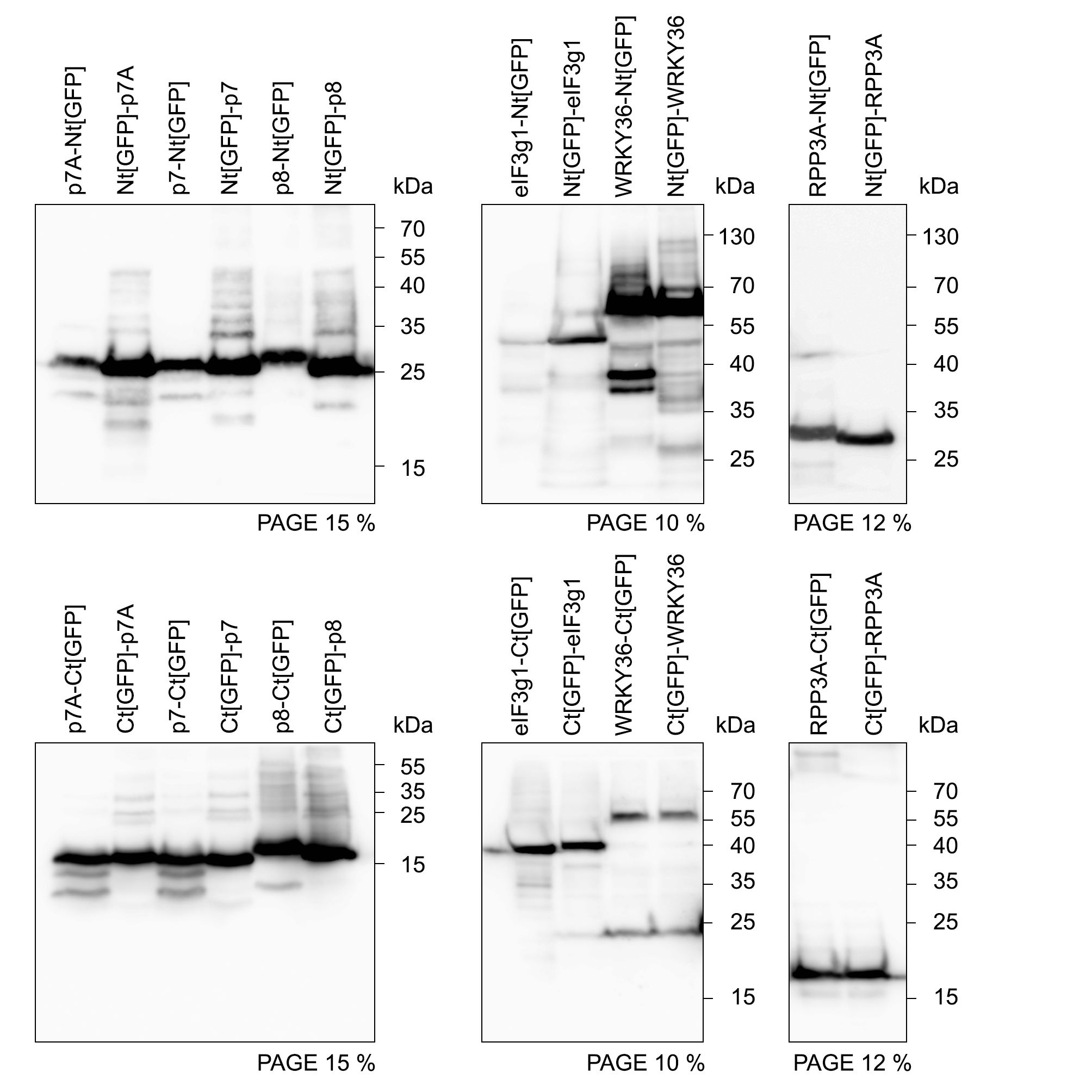
Supplementary Figure S2.** Western Blot analysis of the Nt[GFP]- and Ct[GFP]-labelled proteins used in BiFC assays. The indicated proteins were extracted from 100 mg of fresh tissue using 500 µl of Laemmli buffer and crude extracts clarified by centrifugation. After heat denaturing, 10 µl of each extract were analysed by PAGE at different concentrations, depending on protein molecular weight, and transferred to PVDF membranes. Immunodetection of Nt[GFP]- or Ct[GFP]-tagged proteins was performed using a monoclonal antibody against the Nt or Ct end of the GFP, respectively. Blots were visualized using a quimioluminiscent substrate using a LAS-3000 Imaging System. The positions of the protein molecular weight markers with sizes in kDa are indicated on the right-hand side of each Western blot.


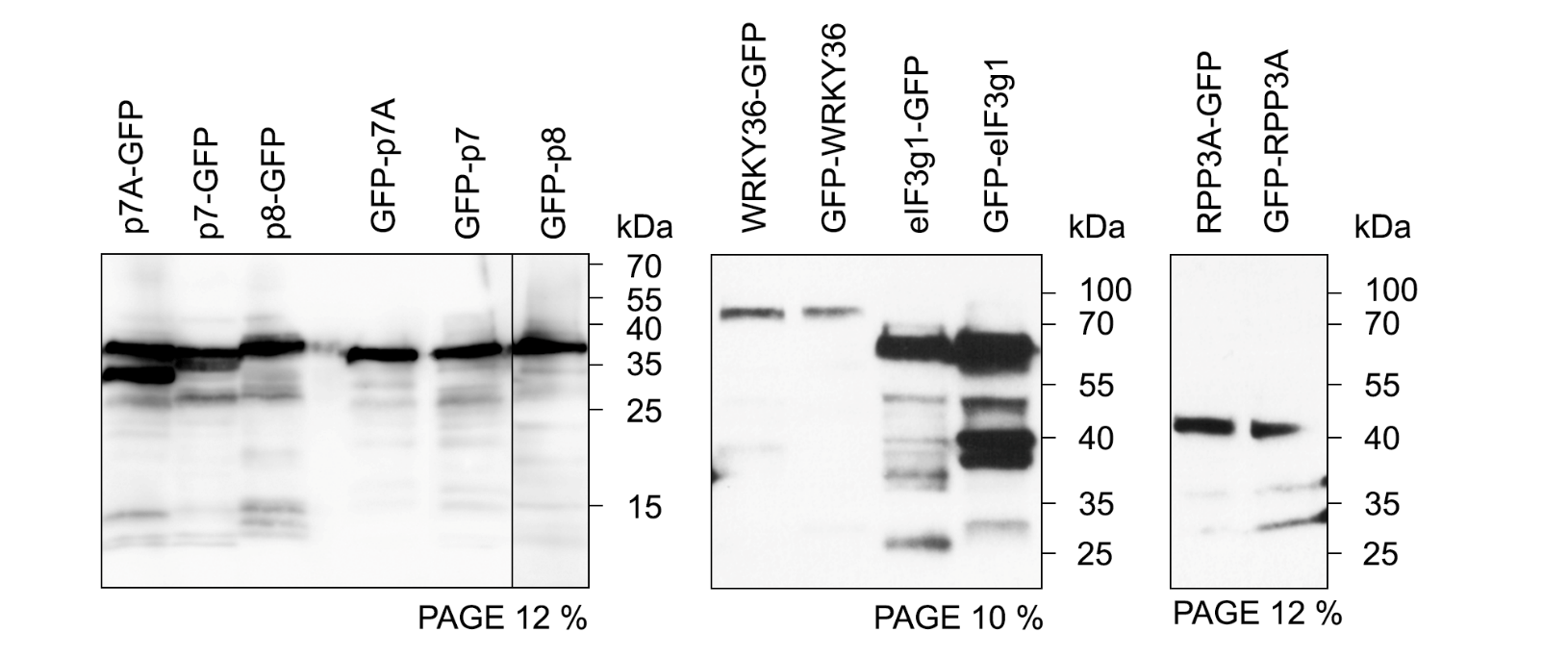
**Supplementary Figure S3.** Western Blot analysis of the GFP-labelled proteins used in subcellular localisation assays. The indicated proteins were extracted from 100 mg of fresh tissue using 500 µl of Laemmli buffer and crude extracts clarified by centrifugation. After heat denaturing, 10 µl of each extract were analysed by PAGE at different concentrations, depending on protein molecular weight, and transferred to PVDF membranes. Immunodetection was performed using a monoclonal antibody against the Ct end of the GFP. Blots were visualized using a quimioluminiscent substrate using a LAS-3000 Imaging System. The positions of the protein molecular weight markers with sizes in kDa are indicated on the right-hand side of each Western blot. The black line between GFP-p7 and GFP-p8 samples in the image on the left indicates different PAGEs.

**
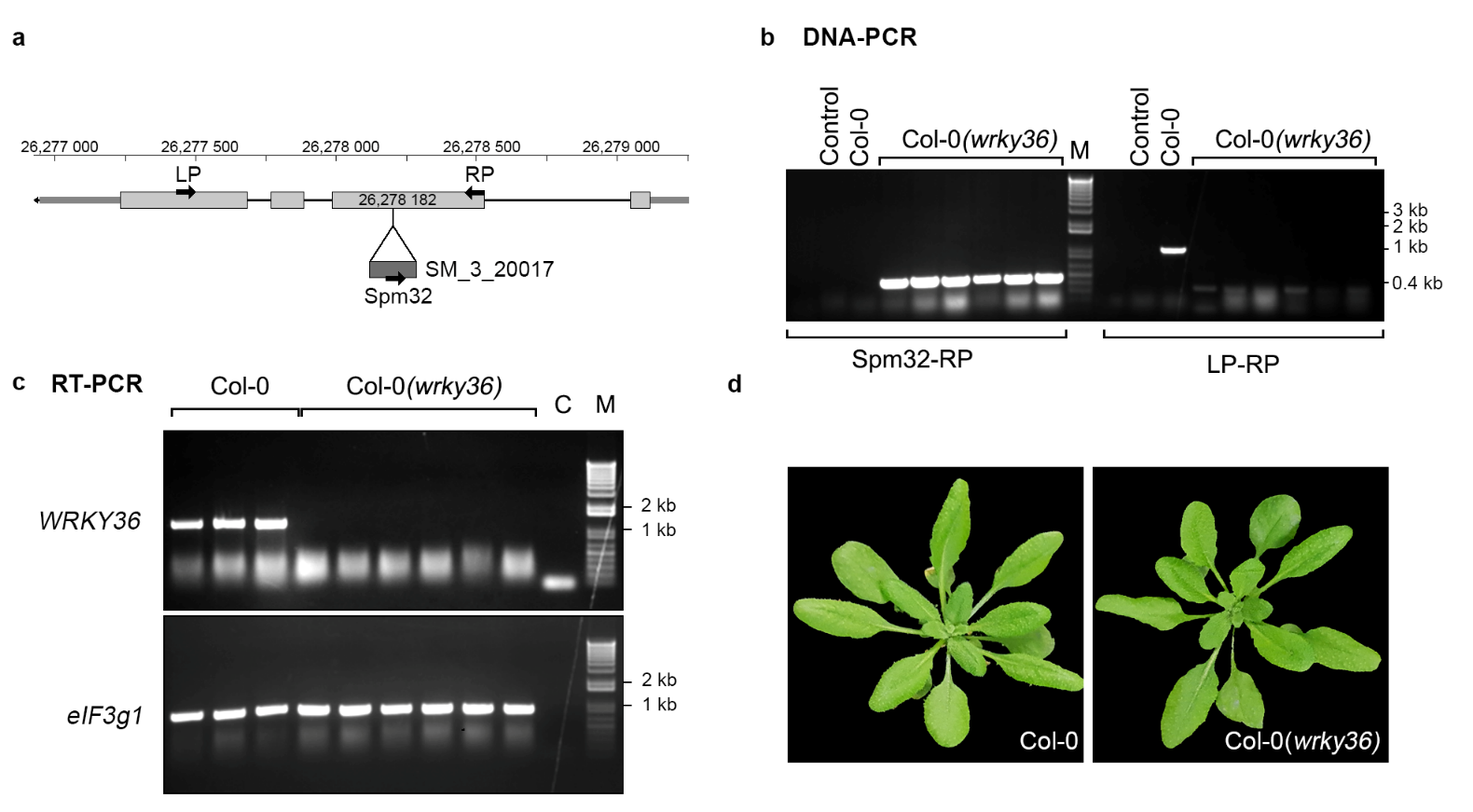
**

**Supplementary Figure S4.** Genotypic and phenotypic characterisation of SM_3_20017 T-DNA insertion mutant (a) Diagram of *WRKY36* structure showing its genomic position at the top, exons (light grey boxes), location of the SM_3_20017 T-DNA insertion (dark grey box) and LP, RP and Spm332 primers for genotyping (arrows). (b) Representative electrophoresis showing confirmation of SM_3_20017 T-DNA insertion in homozygous condition in six Arabidopsis plants. On the DNA marker (M) left, samples correspond to the T-DNA allele and, on the marker right, to the wild-type allele. (c) Agarose gel electrophoresis of RT-PCR products obtained with primers to amplify the full-length *WRKY36* from wild-type Col0 and *wrky36* plants (top panel). Amplification of *eIF3g1* was used as PCR control (bottom panel). C: control without RNA. (d) A representative three-week-old *wrky36* plant (right panel) showing no apparent phenotype compared to wild-type (left panel).


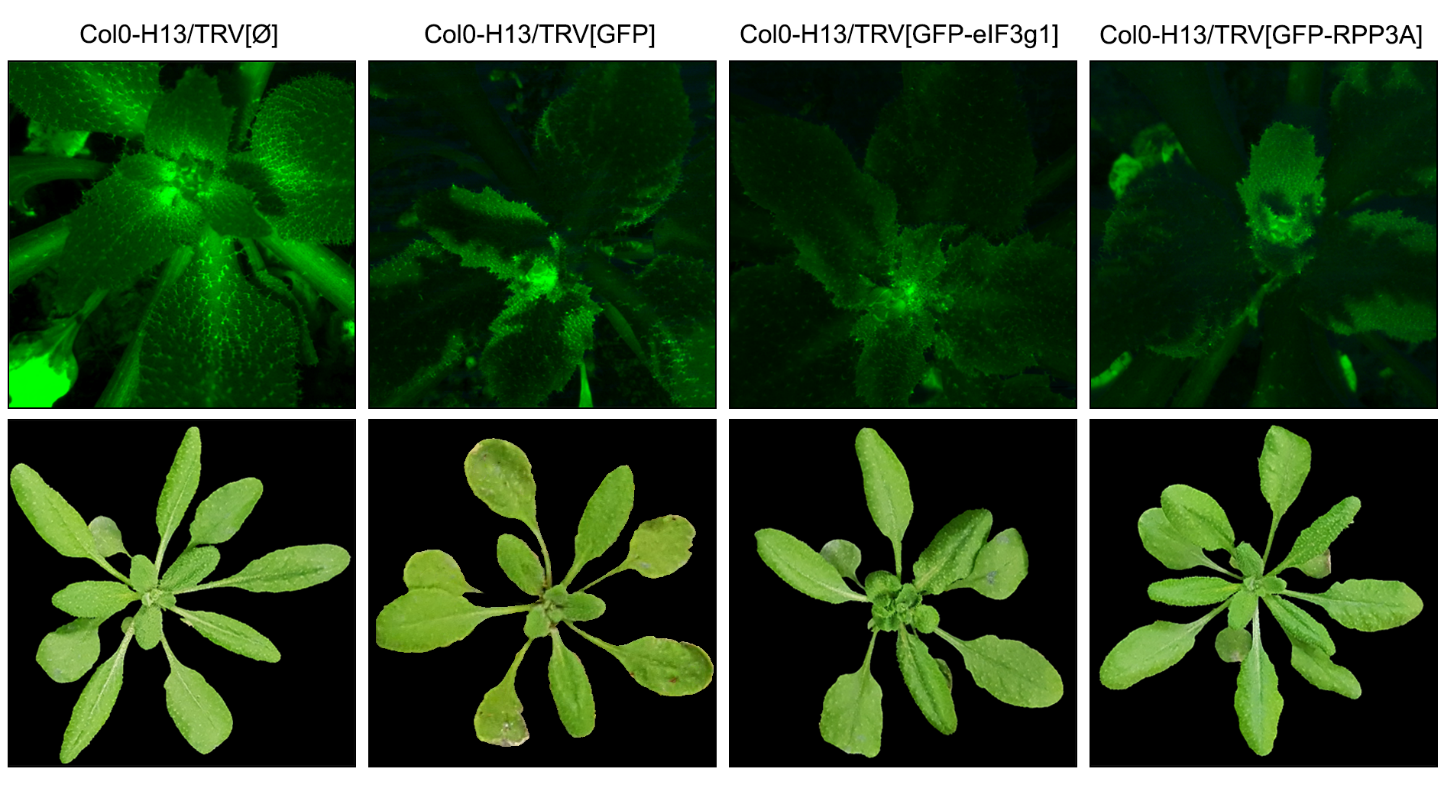


**Supplementary Figure S5.** Phenotype of eIF3G1 and RPP3A-silenced Arabidopsis by TRV-mediated VIGS. Images show GFP-transgenic Arabidopsis plants (Line H13) ten days after infection with TRV1 plus TRV2[Ø], TRV2[GFP], TRV2[GFP-eIF3g1] and TRV2[GFP-RPP3A] taken under UV light (top panels) showing *GFP* silencing, except for TRV2[Ø] infected plant, and under white light (bottom panels) showing no apparent phenotype except for TRV2[GFP-eIF3g1] infected plant.


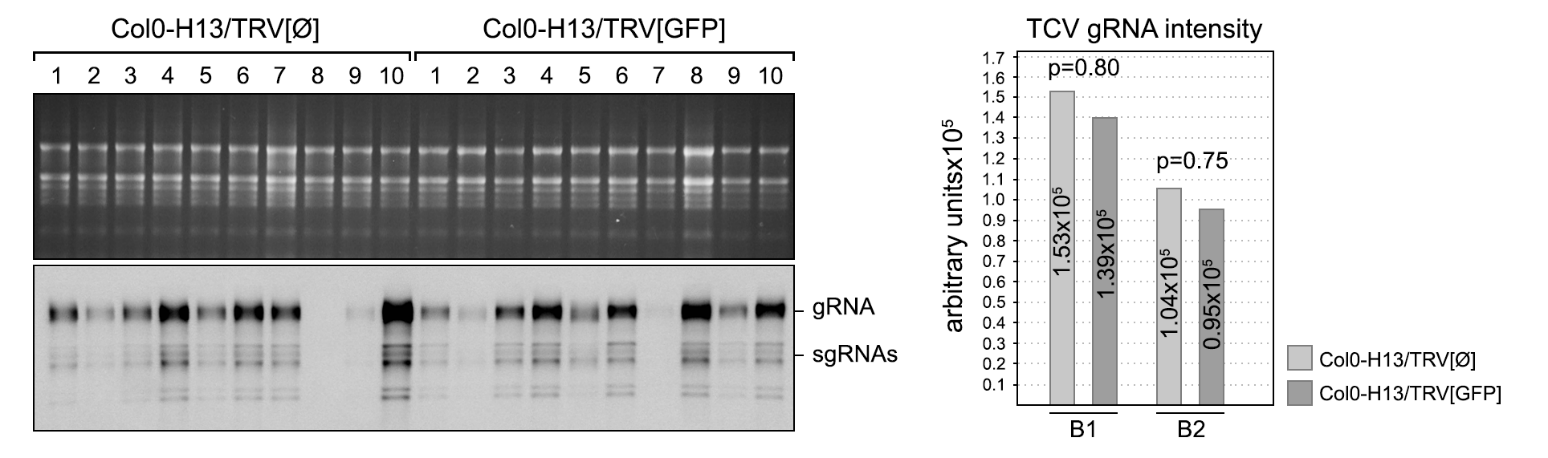


**Supplementary Figure S6.** Comparative analysis of turnip crinkle virus accumulation in Arabidopsis plants infected with TRV[Ø] or TRV[GFP]. The presence of TCV gRNA and sgRNAs was analyzed by Northern blot at 3 dpi (left panels). The results from a representative assay are shown, but three independent experiments including ten plants were performed. Each of the ten RNA samples displayed comes from a unique plant. Ethidium bromide-stained rRNAs serve as loading controls (upper panel). Accumulation of TCV positive-strand gRNA was analysed by quantification of the corresponding hybridisation signal intensity with Image Gauge Software ver. 4.0. Quantification results from two bioassays (B1 and B2) are shown in the bar diagram at the right panel and p values indicated. No significant differences were found.

**BRIEF DESCRIPTION OF THE SUPPLEMENTARY MOVIES**

**Supplementary Movie S1.** Representative 360° 3D reconstruction of fluorescence distribution obtained in BiFC assay with Ct[GFP]-p7A and WRKY36-[GFP] in epidermal cells of *Nicotiana benthamiana* at 48 hpi.

**Supplementary Movie S2**. 360° 3D reconstruction of fluorescence distribution obtained after transient expression of p7A-GFP in epidermal cells of *Nicotiana benthamiana* at 48 hpi.

**Supplementary Movie S3**. 360° 3D reconstruction of fluorescence distribution obtained after transient expression of p7-GFP in epidermal cells of *Nicotiana benthamiana* at 48 hpi.

| Supplementary Table S1. List of DGBp1 interacting proteins. | | | | | | |
| --- | --- | --- | --- | --- | --- | --- |
|  | | | | **Full-length interaction** | | |
| **TAIR access** | **Name** | **Screening identification** | | **p7A** | **p7** | **p8** |
| At1g70790 | Calcium-dependent lipid-binding (CaLB domain) protein | p7A |  | - | - | - |
| At1g23140 | C2-domain ABA-related 8 protein (CAR8) | p7A | p7 | - | - | - |
| At5g40190 | Calmodulin binding protein |  | p7 | - | - | - |
| At1g09070 | Soybean gene regulated by cold-2 (SRC2) | p7A | p7 | - | - | - |
| At5g67360 | Subtilisin-like serine protease (ARA12) | p7A |  | - | - | - |
| At5g15090 | Voltage dependent anion channel 3 (VDAC3) | p7A | p7 | - | - | - |
| At1g04870 | Protein arginine methyltransferase 10 (PRMT10) | p7A | p7 | - | - | - |
| At3g05010 | G-protein coupled receptor 2, (CAND2) | p7A | p7 | - | - | - |
| At1g18310 | Glycosyl hydrolase family 81 protein | p7A |  | - | - | - |
| At1g10350 | DNA-J heat shock family protein | p7A | p7 | - | - | - |
| At5g02500 | Heat shock cognate protein 70-1 | p7A |  | - | - | - |
| At5g02480 | HSP20-like chaperones superfamily protein |  | p7 | - | - | - |
| At5g48030 | Gametophitic factor 2 (GFA2); DNAJ family protein |  | p7 | - | - | - |
| At3g09440 | Heat shock protein 70 (Hsp70) |  | p7 | - | - | - |
| At5g11170 | Dead-box ATP-dependent RNA-helicase 56 |  | p7 | - | - | - |
| At5g62190 | Dead-box ATP-dependent RNA helicase 7 (PRH75) |  | p7 | - | - | - |
| At2g26460 | RNA splicing protein (SMU2) | p7A |  | - | - | - |
| At5g11200 | ATP-dependent RNA helicase (UAP56B) | p7A |  | - | - | - |
| At4g05320 | Polyubiquitin 10 | p7A |  | - | - | - |
| At2g35330 | RING/U-box superfamily protein |  | p7 | - | - | - |
| At2g35635 | NEDD8-like protein RUB2 (UBQ7) |  | p7 | - | - | - |
| At2g40640 | RING/U-box superfamily protein |  | p7 | - | - | - |
| At3g48070 | RING/U-box superfamily protein |  | p7 | - | + | - |
| At5g42220 | Ubiquitin-like superfamily protein |  | p7 | - | - | - |
| At3g52590 | Ubiquitin extension protein (UBQ1) |  | p7 | - | - | - |
| At4g25890 | 60S acidic ribosomal protein (RPP3A) | p7A | p7 | + | + | + |
| At3g11400 | G subunit of eukaryotic initiation factor 3 (EIF3g). | p7A | p7 | + | + | + |
| At3g45030 | Ribosomal protein S10p/S20e family protein | p7A | p7 | - | - | - |
| At1g72370 | 40S Ribosomal protein SA |  | p7 | - | - | - |
| At5g62300 | Ribosomal protein S10p/S20e family protein |  | p7 | - | - | - |
| At3g02550 | LOB domain-containing protein 41 (LBD41) | p7A |  | + | + | - |
| At1g69810 | Transcription factor WRKY36 | p7A | p7 | + | + | + |
| At1g43890 | RAB GTPase homolog 18 (ATRAB-18) |  | p7 | - | - | - |
| At3g59470 | FAR1-related sequences-related factor1 (FRF1) |  | p7 | - | - | - |
| At5g19090 | Heavy metal transport/detoxification protein |  | p7 | - | - | - |
| At5g41600 | Reticulon like protein B4 (RTNLB4) |  | p7 | - | - | - |
| At1g48580 | Uncharacterised protein |  | p7 | - | - | - |
| At1g21740 | Uncharacterised protein |  | p7 | - | - | - |
| At1g20100 | DNA ligase-like protein |  | p7 | - | + | + |
| At1g48580 | Nuclear localised protein |  | p7 | - | + | - |
| At1g14630 | Uncharacterised protein |  | p7 | - | - | - |

| Supplementary Table S2. Bimolecular fluorescence complementation results | | | | | | |
| --- | --- | --- | --- | --- | --- | --- |
|  | **eIF3g1** | | **RPP3A** | | **WRKY36** | |
| **p7A** | Ct[GFP]-p7A+Nt[GFP]-eIF3g1 | - | Ct[GFP]-p7A+Nt[GFP]-RPP3A | - | Ct[GFP]-p7A+Nt[GFP]-WRKY36 | - |
|  | Ct[GFP]-p7A+eIF3g1-Nt[GFP] | ++ | Ct[GFP]-p7A+RPP3A-Nt[GFP] | ++ | Ct[GFP]-p7A+WRKY36-Nt[GFP] | ++ |
|  | p7A-Ct[GFP]+Nt[GFP]-eIF3g1 | - | p7A-Ct[GFP]+Nt[GFP]-RPP3A | - | p7A-Ct[GFP]+Nt[GFP]-WRKY36 | - |
|  | p7A-Ct[GFP]+eIF3g1-Nt[GFP] | - | p7A-Ct[GFP]+RPP3A-Nt[GFP] | - | p7A-Ct[GFP]+WRKY36-Nt[GFP] | - |
|  | Nt[GFP]-p7A+Ct[GFP]-eIF3g1 | - | Nt[GFP]-p7A+Ct[GFP]-RPP3A | - | Nt[GFP]-p7A+Ct[GFP]-WRKY36 | - |
|  | Nt[GFP]-p7A+eIF3g1-Ct[GFP] | + | Nt[GFP]-p7A+RPP3A-Ct[GFP] | + | Nt[GFP]-p7A+WRKY36-Ct[GFP] | + |
|  | p7A-Nt[GFP]+Ct[GFP]-eIF3g1 | - | p7A-Nt[GFP]+Nt[GFP]-CPP3A | - | p7A-Nt[GFP]+Ct[GFP]-WRKY36 | - |
|  | p7A-Nt[GFP]+eIF3g1-Ct[GFP] | - | p7A-Nt[GFP]+RPP3A-Ct[GFP] | - | p7A-Nt[GFP]+WRKY36-Ct[GFP] | - |
| **p7** | Ct[GFP]-p7+Nt[GFP]-eIF3g1 | - | Ct[GFP]-p7+Nt[GFP]-RPP3A | - | Ct[GFP]-p7+Nt[GFP]-WRKY36 | - |
|  | Ct[GFP]-p7+eIF3g1-Nt[GFP] | ++ | Ct[GFP]-p7+RPP3A-Nt[GFP] | ++ | Ct[GFP]-p7+WRKY36-Nt[GFP] | ++ |
|  | p7-Ct[GFP]+Nt[GFP]-eIF3g1 | - | p7-Ct[GFP]+Nt[GFP]-RPP3A | - | p7-Ct[GFP]+Nt[GFP]-WRKY36 | - |
|  | p7-Ct[GFP]+eIF3g1-Nt[GFP] | - | p7-Ct[GFP]+RPP3A-Nt[GFP] | - | p7-Ct[GFP]+WRKY36-Nt[GFP] | - |
|  | Nt[GFP]-p7+Ct[GFP]-eIF3g1 | - | Nt[GFP]-p7+Ct[GFP]-RPP3A | - | Nt[GFP]-p7+Ct[GFP]-WRKY36 | - |
|  | Nt[GFP]-p7+eIF3g1-CNt[GFP] | + | Nt[GFP]-p7+RPP3A-Ct[GFP] | + | Nt[GFP]-p7+WRKY36-Ct[GFP] | + |
|  | p7-Nt[GFP]+Ct[GFP]-eIF3g1 | - | p7-Nt[GFP]+Ct[GFP]-RPP3A | - | p7-Nt[GFP]+Ct[GFP]-WRKY36 | - |
|  | p7-Nt[GFP]+eIF3g1-Ct[GFP] | - | p7-Nt[GFP]+RPP3A-Ct[GFP] | - | p7-Nt[GFP]+WRKY36-Ct[GFP] | - |
| **p8** | Ct[GFP]-p8+Nt[GFP]-eIF3g1 | - | Ct[GFP]-p8+Nt[GFP]-RPP3A | - | Ct[GFP]-p8+Nt[GFP]-WRKY36 | - |
|  | Ct[GFP]-p8+eIF3g1-Nt[GFP] | ++ | Ct[GFP]-p8+RPP3A-Nt[GFP] | ++ | Ct[GFP]-p8+WRKY36-Nt[GFP] | ++ |
|  | p8-Ct[GFP]+Nt[GFP]-eIF3g1 | - | p8-Ct[GFP]+Nt[GFP]-RPP3A | - | p8-Ct[GFP]+Nt[GFP]-WRKY36 | - |
|  | p8-Ct[GFP]+eIF3g1-Nt[GFP] | - | p8-Ct[GFP]+RPP3A-Nt[GFP] | - | p8-Ct[GFP]+WRKY36-Nt[GFP] | - |
|  | Nt[GFP]-p8+Ct[GFP]-eIF3g1 | - | Nt[GFP]-p8+Ct[GFP]-RPP3A | - | Nt[GFP]-p8+Ct[GFP]-WRKY36 | - |
|  | Nt[GFP]-p8+eIF3g1-Ct[GFP] | + | Nt[GFP]-p8+RPP3A-Ct[GFP] | + | Nt[GFP]-p8+WRKY36-Ct[GFP] | + |
|  | p8-Nt[GFP]+Ct[GFP]-eIF3g1 | - | p8-Nt[GFP]+Ct[GFP]-RPP3A | - | p8-Nt[GFP]+Ct[GFP]-WRKY36 | - |
|  | p8-Nt[GFP]+eIF3g1-Ct[GFP] | - | p8-Nt[GFP]+RPP3A-Ct[GFP] | - | p8-Nt[GFP]+WRKY36-Ct[GFP] | - |
|  | **Negative controls** | | | | | |
|  | **MNSV p7A** | | **CarMV p7** | | **TCV p8** | |
|  | Ct[GFP]-p7A+Nt[GFP] | - | Ct[GFP]-p7+Nt[GFP] | - | Ct[GFP]-p8+Nt[GFP] | - |
|  | p7A-Ct[GFP]+Nt[GFP] | - | p7-Ct[GFP]+Nt[GFP] | - | P8-Ct[GFP]+Nt[GFP] | - |
|  | Nt[GFP]-p7A+Ct[GFP] | - | Nt[GFP]-p7+Ct[GFP] | - | Nt[GFP]-p8+Ct[GFP] | - |
|  | p7A-Nt[GFP]+Ct[GFP] | - | p7-Nt[GFP]+Ct[GFP] | - | P8-Nt[GFP]+Ct[GFP] | - |

| SupplementaryTable S3. List of oligonucleotides used in this study. | | | |
| --- | --- | --- | --- |
| Oligonucleotides used for molecular cloning | | | |
| Plasmid | Insert | Forward primer | Reverse primer |
| pGBKT7 | MNSV p7A | ACGTAAGCTTCCATGGACTCTCAACGAACT NcoI^1^ | ACGTGGATCCCTAAAAGTTAAAGTTAA BamHI |
| pGBKT7 | CarMV p7 | AGCTCCATGGATATTGAATCGGAAG NcoI | AGCTTGGATCCCTAAAAGTTGAAGTGAATGTG BamHI |
| pGADT7 | MNSV p7A | ACGTAAGCTTCCATGGACTCTCAACGAACT NcoI | ACGTGGATCCCTAAAAGTTAAAGTTAA BamHI |
| pGADT7 | CarMV p7 | AGCTCCATGGATATTGAATCGGAAG NcoI | AGCTGGATCCCTAAAAGTTGAAGTGAATGTG BamHI |
| pGBKT7 | TCV p8 | ACGTCCATGGATCCTGAACGAATTC NcoI | ACGTCTGCAGTTAGAAGTTGAAGTTGATTG PstI |
| pGBKT7 | MNSV p7A^Δ1-22^ | GGGGGAAAACAGAAGAACTCAATG | GGCCATATGCAGGTCCTCCTC |
| pGBKT7 | MNSV p7A^Δ23-44^ | GGAGTTATGGGTGCTAGCAC | GCTGTCACCACGTTCTTTAC |
| pGBKT7 | MNSV p7A ^Δ45-65^ | TAGGGATCCGTCGACCTGCAG | TTGCTTCGATTCAGAGATAGCATC |
| pGBKT7 | MNSV p7A^fnf^ | ACGTAAGCTTCCATGGACTCTCAACGAACT NcoI | ACGTGGATCCCTAAGCGGCAGCGTTAATAGTCACCTTAAT BamHI |
| pGBKT7 | CarMV p7^fnf^ | AGCTCCATGGATATTGAATCGGAAG NcoI | AGCTGGATCCCTAAGCGGCGGCGTGAATGTGCACCTC BamHI |
| pGBKT7 | TCV p8^fnf^ | ACGTCCATGGATCCTGAACGAATTC NcoI | ACGTCTGCAGTTAAGCAGCAGCGTTGATTGAGACTTCCACTTTATC PstI |
| pGBKT7 | At3g11400 | AGCTCATATGACGATCGATTCGCAGCAA NdeI | ACGTGGATCCCTAGGTTGGTCTTGGAGT BamHI |
| pGBKT7 | At4g25890 | AGCTCATATGGGAGTATTCACATTCGTA NdeI | ACGTGGATCCTTAACCAAAGAGATCGAA BamHI |
| pGBKT7 | At1g69810 | AGCTCATATGATCAAAGAGGAGACCGTT NdeI | AGCTGGATCCTTATTGCTGTCCGGAAAG BamHI |
| pGBKT7 | At5g06000 | ACGTCATATGGCGATTGATACGATA NdeI | ACGTGGATCCTCATGAATGACATATATTC BamHI |
| pGBKT7 | At5g57290 | ACGTCATATGGGAGTTTTCTCATTCGTGTGCAAAAGC NdeI | ACGTGGATCCTTAACCAAAGAGATCAAATCCAAAGTCTCCC BamHI |
| pMOG800 | eIF3g1-Nt/Ct[YFP], -ChFP | AGCTTCATGACGATCGATTCGCAGCAA PagI | ACGTTCTAGAGGTTGGTCTTGGAGT XbaI |
| pMOG800 | Nt/Ct[YFP]-,ChFP-eIF3g1 | AGCTTCATGACGATCGATTCGCAGCAA PagI | ACGTTCTAGACTAGGTTGGTCTTGGAGT XbaI |
| pMOG800 | WRKY36-Nt/Ct[YFP], -ChFP | AGCTACATGTTGAAAGAGGAGACCGTT PciI | ACGTGCTAGCTTGCTGTCCGGAAAG NheI |
| pMOG800 | Nt/Ct[YFP]-, ChFP-WRKY36 | AGCTACATGTTGAAAGAGGAGACCGTT PciI | ACGTGCTAGCTTATTGCTGTCCGGAAAG NheI |
| pMOG800 | RPP3A-Nt/Ct[YFP], -ChFP | AGCTCCATGGGAGTATTCACATTCGTA NcoI | ACGTGCTAGCACCAAAGAGATCGAA NheI |
| pMOG800 | Nt/Ct[YFP]-, ChFP-RPP3A | AGCTCCATGGGAGTATTCACATTCGTA NcoI | ACGTGCTAGCTTAACCAAAGAGATCGAA NheI |
| pMOG800 | p7A-Nt/Ct[YFP] | ACGTAAGCTTCCATGGACTCTCAACGAACT NcoI | ACGTTCTAGAAAAGTTAAAGTTAA XbaI |
| pMOG800 | Nt/Ct[YFP]/p7A | ACGTAAGCTTCCATGGACTCTCAACGAACT NcoI | ACGTTCTAGACTAAAAGTTAAAGTTAA XbaI |
| pMOG800 | p7-Nt/Ct[YFP]  p7-GFP | AGCTCCATGGATATTGAATCGGAAGTA NcoI | ACGTGCTAGCAAAGTTGAAGTG NheI |
| pMOG800 | Nt/Ct[YFP]-p7  GFP-p7 | AGCTCCATGGATATTGAATCGGAAGTA NcoI | ACGTGCTAGCCTAAAAGTTGAAGTG NheI |
| pMOG800 | p8-Nt/Ct[YFP]  p8-GFP | ACGTCCATGGATCCTGAACGAATTC NcoI | ACGTTCTAGAGAAGTTGAAGTTGATTGAGAC XbaI |
| pMOG800 | Nt/Ct[YFP]-p8  GFP-p8 | ACGTCCATGGATCCTGAACGAATTC NcoI | ACGTTCTAGATTAGAAGTTGAAGTTGATTGAGAC XbaI |
| pDONR207/pTRV2 | Partial mGFP5 | *GGGGACAAGTTTGTACAAAAAAGCAGGCTTC*AGTAAAGGAGAAGAACTTTTC attB1^2^ | **CCTTGAAGAAGATGGTCCTCTC**^3^ |
| pDONR207/pTRV2 | eIF3g1 | **GAGAGGACCATCTTCTTCAAGG**ATGACGATCGATTCGCAGC | *GGGGACCACTTTGTACAAGAAAGCTGGGTG*AGAAACCATAGTGAGATGGCTAC attB2 |
| pDONR207/pTRV2 | RPP3A | **GAGAGGACCATCTTCTTCAAGG**ATGGGAGTATTCACATTCGTATG | *GGGGACCACTTTGTACAAGAAAGCTGGGTG*ACCTGGAATACGGCGGAGG attB2 |
| pDONR207/pTRV2 | Full mGFP5 | *GGGGACAAGTTTGTACAAAAAAGCAGGCTTC*AGTAAAGGAGAAGAACTTTTC attB1 | *GGGGACCACTTTGTACAAGAAAGCTGGGTG*TTTGTATAGTTCATCCATGCC attB2 |
| Oligonucleotides used for Real Time RT-qPCR | | | |
| Gene | Access | Forward primer | Reverse primer |
| At EF1 | At5g60390 | TGGTGACGCTGGTATGGTTA | TCCTTCTTGTCCACGCTCTT |
| At F-BOX | At5g15710 | GGCTGAGAGGTTCGAGTGTT | GGCTGTTGCATGACTGAAGA |
| At PDF2 | At1g13320 | GGCAGAAGTTCGGATAGCAG | CAATGCAGATCTGACGTGCT |
| At eIF3g1 | At3g11400 | TGCGTGTAACCAATCTGTCCGAG | CCAAATCCTCTGCTCACTCCA |
| At RPP3A | At4g25890 | CTGGAGGAGCTGCCTCAAGT | AAAGAGATCGAATCCGAAGTCTCC |
| TCV p28 | NC_003821 | TCGTGCTGGATTTGCACAGTG | CATAATCGCCTCCAAGCCCTG |
| **Oligonucleotides used for site-directed mutagenesis** | | | |
| Plasmid | gene | Forward primer | Reverse primer |
| PZP-TCV-sGFP/ pTCV-M | p8 | GGAAGTCTCAATCAACGTCAACGTCTAATCAGACATGTCAG | CTGACATGTCTGATTAGACGTTGACGTTGATTGAGACTTCC |
| ^1^ restriction enzymes used are indicated and their restriction sites underlined.  ^2^ Gateway recombination sequences attB1 and attB2 are shown in italic.  ^3^ Overlapping sequence is shown in bold. | | | |
